# Eco-evolutionary model on spatial graphs reveals how habitat structure affects phenotypic differentiation

**DOI:** 10.1038/s42003-022-03595-3

**Published:** 2022-07-06

**Authors:** Victor Boussange, Loïc Pellissier

**Affiliations:** 1 Swiss Federal Research Institute WSL, CH-8903 Birmensdorf, Switzerland; 2 Landscape Ecology, Institute of Terrestrial Ecosystems, Department of Environmental Systems Science, ETH Zürich, CH-8092 Zürich, Switzerland

**Keywords:** Network topology, Population dynamics, Biodiversity, Evolutionary ecology

## Abstract

Differentiation mechanisms are influenced by the properties of the landscape over which individuals interact, disperse and evolve. Here, we investigate how habitat connectivity and habitat heterogeneity affect phenotypic differentiation by formulating a stochastic eco-evolutionary model where individuals are structured over a spatial graph. We combine analytical insights into the eco-evolutionary dynamics with numerical simulations to understand how the graph topology and the spatial distribution of habitat types affect differentiation. We show that not only low connectivity but also heterogeneity in connectivity promotes neutral differentiation, due to increased competition in highly connected vertices. Habitat assortativity, a measure of habitat spatial auto-correlation in graphs, additionally drives differentiation under habitat-dependent selection. While assortative graphs systematically amplify adaptive differentiation, they can foster or depress neutral differentiation depending on the migration regime. By formalising the eco-evolutionary and spatial dynamics of biological populations on graphs, our study establishes fundamental links between landscape features and phenotypic differentiation.

## Abstract (web summary)

Analytical methods and numerical simulations on graph representations of landscapes reveal how habitat connectivity and heterogeneity affect phenotypic differentiation.

## Introduction

Biodiversity results from differentiation processes influenced by the features of the landscape over which populations are distributed^1^. The documentation of high levels of species diversity in mountain regions and riverine systems suggests that complex connectivity patterns and habitat heterogeneity foster differentiation^2–5^. However, hypotheses formulated based on empirical evidence should be complemented by mechanistic models to crystallise a causal understanding between processes and patterns^6^. While the number of simulation studies is growing steadily^7^, such studies often lack a mathematical formalism to facilitate the interpretation of the model outcomes by providing an analytical underpinning to the simulation results^8^.

Phenotypic differentiation processes emerge as a result of mutation, selection, and migration and can be classified as neutral or adaptive^9^. Neutral differentiation is initiated by the stochastic drift of local phenotypes when spatial isolation and limited dispersal create barriers to gene flow, allowing distinct phenotypes to emerge in spatially structured populations^10^. In contrast, adaptive differentiation results from heterogeneous selection, which promotes distinct, locally well-adapted phenotypes in populations occupying patches with different habitat conditions^11^. The evolution of neutral phenotypes and of adaptive phenotypes are not independent, as selective forces can indirectly select for those neutral phenotypes that happen to be linked to the fittest adaptive phenotypes, a mechanism called the “hitchhiking effect”^12^. Moreover, selection can generate barriers to gene flow between populations in heterogeneous habitat landscapes^13,14^, a phenomenon coined “isolation by environment”, which can amplify neutral differentiation. How neutral processes, adaptive processes, and their interplay are affected by landscape features is difficult to comprehend without a formalised mechanistic model^15^.

Models link patterns to processes^6^, and the explicit representation of the landscape within an eco-evolutionary model can lead to a causal understanding of how landscape features shape differentiation. Spatial graphs provide a convenient mathematical representation of landscapes, where vertices represent suitable habitats hosting populations, and edges capture the connectivity between habitats^16^. Under ecological dynamics, metapopulation models have been used to study the role of graph topology in the persistence and stability of metapopulation^17–20^ and community diversity^21–23^. Evolutionary mechanisms are nevertheless fundamental drivers of diversity, and should therefore be explicitly integrated into models^24^. Evolutionary game theory explores how graph topology impacts the fixation probability and the fixation time of a mutated phenotype^25^. However, the framework does not consider the continuous accumulation of mutations, and is therefore not suited to addressing the emergence of phenotypic differentiation. By combining a metapopulation model with a model of neutral evolution,^26,27^ investigated how graph topology affects neutral diversity. Their approach demonstrated the key role of topological properties in shaping diversity, and its predictions could be matched with empirical data from e.g., river basins^28^. Nonetheless, diversity results from the combination of neutral and adaptive processes developing at the population level. A first-principles modelling approach considering spatial graphs, but also building upon the elementary processes of ecological interactions, reproduction, mutation, and migration may therefore be promising to investigate the emergence of diversity.

Stochastic models for structured populations, rooted in the microscopic description of individuals, offer a generic framework for modelling eco-evolutionary dynamics^29,30^. In particular, these models can capture the interplay between population dynamics, spatial dynamics and phenotypic evolution, while providing a rigorous set-up for analytical investigation. By anchoring this modelling paradigm in a mathematical framework, the work of Champagnat et al.^29^ generalises models of population genetics^31^ (investigating the evolution of the frequencies of alleles) and quantitative genetics^32–34^ (investigating the evolution of phenotypic traits), which stimulated research into the link between spatial population structure and neutral differentiation. The framework embraces density-dependent selection, which could explain the emergence of phenotypic differentiation from competition processes^11^, and how spatial segregation can emerge as a byproduct of these adaptive processes along environmental gradients^35^. Related models have addressed the effects of landscape dynamics and habitat heterogeneity on adaptive differentiation, providing mathematical insights into the dynamics^36–41^. Because it accounts for finite population size, the baseline model of Champagnat et al.^29^ can also capture neutral differentiation dynamics and therefore the coupling between neutral and adaptive processes^42,43^. Nonetheless, the aforementioned studies were not spatially explicit^42,43^ or they assumed regular spatial structures (regular graphs^36–38,41^ or continuous space^35,39,40^), therefore not addressing the role of the spatial complexity of landscapes. A stochastic individual-based model using spatial graphs as a representation of the landscape could help formalise fundamental links between landscape features and phenotypic differentiation.

A key challenge is to understand how individual dynamics result in the emergence of differentiation in complex landscapes^44^. Here, we investigate how complex connectivity patterns and habitat heterogeneity affect both neutral and adaptive phenotypic differentiation by constructing an individual-based model (IBM) that accounts for eco-evolutionary dynamics on spatial graphs. The individuals disperse between habitat patches and possess co-evolving neutral and adaptive traits. The finite size of local populations generates neutral differentiation by inducing a stochastic drift in the neutral trait evolution, while heterogeneous selection gives rise to adaptive differentiation. Macroscopic properties of the model are analytically tractable, and we obtain a deterministic approximation of population size and adaptive trait dynamics which connects the emerging patterns to the graph properties that generate them. However, neutral differentiation is stochastic by nature, which complicates its analytical underpinning. We therefore rely on numerical simulations of the IBM to measure the effect of graph topology on neutral differentiation. In the case where heterogeneous selection is absent, we investigate how graph topology affects neutral differentiation. In the case of heterogeneous selection, we investigate how the graph topology, in combination with the spatial distribution of habitat types, affects levels of (i) adaptive and (ii) neutral differentiation. By combining analytical methods with numerical simulations, we expect to identify graph properties that determine the level of differentiation. Overall, our study establishes causal links between landscape properties and population differentiation and contributes to a fundamental understanding of how landscape features promote biodiversity.

## Results

### Eco-evolutionary model on spatial graphs

We establish an individual-based model (IBM) where individuals are structured over a trait space and a graph representing a landscape. For the sake of simplicity, we consider the case of asexual reproduction and haploid genetics^29^. Individuals die, reproduce, mutate and migrate in a stochastic fashion, which together results in macroscopic properties. The formulation of the stochastic IBM allows an analytical description of the dynamics at the population level, which links emergent properties to the elementary processes that generate them.

The trait space ${{{{{{{\mathcal{X}}}}}}}}\subseteq {{\mathbb{R}}}^{d}$ is continuous and can be split into a neutral trait space ${{{{{{{\mathcal{U}}}}}}}}$ and an adaptive trait space ${{{{{{{\mathcal{S}}}}}}}}$. We refer to neutral traits $u\in {{{{{{{\mathcal{U}}}}}}}}$ as traits that are not under selection, in contrast to adaptive traits $s\in {{{{{{{\mathcal{S}}}}}}}}$, which experience selection. The graph denoted by *G* is composed of a set of vertices {*v*_1_,*v*_2_,…,*v*_*M*_} that correspond to habitat patches (suitable geographical areas), and a set of edges that constrain the movement of individuals between the habitat patches. We use the original measure of genetic differentiation for quantitative traits *Q*_*ST*_ (standing for *Q*-statistics) in the case of haploid populations^45,46^. We denote the neutral trait value of the *k*th individual on *v*_*i*_ as ${u}_{k}^{(i)}$, the number of individuals on *v*_*i*_ as *N*^(*i*)^, the mean neutral trait on *v*_*i*_ as ${\overline{u}}^{(i)}$, and the mean neutral trait in the metapopulation as $\overline{u}$. It follows that we quantify neutral differentiation *Q*_*ST*,*u*_ as

1 $${Q}_{ST,u}={\sigma }_{B,u}^{2}/({\sigma }_{B,u}^{2}+{\sigma }_{W,u}^{2})$$

where ${\sigma }_{B,u}^{2}={\mathbb{E}}[\frac{1}{M}{\sum }_{i}{\left({\overline{u}}^{(i)}-\overline{u}\right)}^{2}]$ denotes the expected neutral trait variance between the vertices and ${\sigma }_{W,u}^{2}=\frac{1}{M}\mathop{\sum }\nolimits_{i}^{M}{\mathbb{E}}\left[\frac{1}{{N}^{(i)}}{\sum }_{k}{\left({u}_{k}^{(i)}-{\overline{u}}^{(i)}\right)}^{2}\right]$ denotes the average expected neutral trait variance within vertices. We similarly quantify adaptive differentiation *Q*_*ST*,*s*_.

**Fig. 1:**
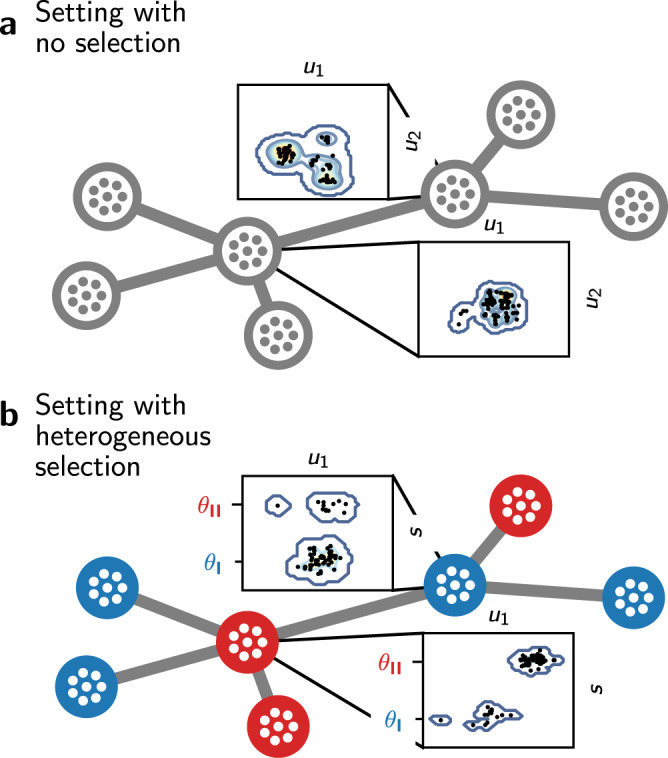
Graphical representation of the structure of individuals in the eco-evolutionary model. **a** Setting with no selection, where individuals are characterised by a set of neutral traits $u\in {{{{{{{\mathcal{U}}}}}}}}$. The scatter plots represent a projection of the first two components of *u* for the individuals present on the designated vertices at time *t* = 1000, obtained from one simulation of the IBM. **b** Setting with heterogeneous selection. In this setting, individuals are additionally characterised by adaptive traits $s\in {{{{{{{\mathcal{S}}}}}}}}$. Blue vertices favour the optimal adaptive trait value *θ*_**I**_, while red vertices favour *θ*_**II**_. The scatter plots represent a projection of the first component of *u* and *s* for the individuals present on the designated vertices at time *t* = 1000, obtained from one simulation. The majority of individuals are locally well-adapted and have an adaptive trait close to the optimal value, but some maladaptive individuals originating from neighbouring vertices are also present. *m* = 0.05.

Following the Gillespie update rule^47^, individuals with trait ${x}_{k}\in {{{{{{{\mathcal{X}}}}}}}}$ on vertex *v*_*i*_ are randomly selected to give birth at rate *b*^(*i*)^(*x*_*k*_) and die at rate *d*(*N*^(*i*)^) = *N*^(*i*)^/*K*, where *K* is the local carrying capacity. The definition of *d* therefore captures competition, which is proportional to the number of individuals on a vertex and does not depend on the individuals’ traits (we relax this assumption later on). The offspring resulting from a birth event inherits the parental traits, which can independently be affected by mutations with probability *μ*. A mutated trait differs from the parental trait by a random change that follows a normal distribution with variance ${\sigma }_{\mu }^{2}$ (corresponding to the continuum of alleles model^48^). The offspring can further migrate to neighbouring vertices by executing a simple random walk on *G* with probability *m*. A schematic overview of the two different settings considered is provided in Fig. 1. Under the setting with no selection, individuals are only characterised by neutral traits so that ${{{{{{{\mathcal{X}}}}}}}}={{{{{{{\mathcal{U}}}}}}}}$. For individuals on a vertex with trait *x*_*k*_ ≡ *u*_*k*_ we define *b*^(*i*)^(*x*_*k*_) ≡ *b*, so that the birth rate is constant. This ensures that neutral traits do not provide any selective advantage. Under the setting with heterogeneous selection, each vertex of the graph *v*_*i*_ is labelled by a habitat type with environmental condition Θ_*i*_ that specifies the optimal adaptive trait value on *v*_*i*_. It follows that, for individuals with traits ${x}_{k}=({u}_{k},{s}_{k})\in {{{{{{{\mathcal{U}}}}}}}}\times {{{{{{{\mathcal{S}}}}}}}}$ on *v*_*i*_, we define

2 $${b}^{(i)}({x}_{k})\equiv {b}^{(i)}({s}_{k})=b(1-p{({s}_{k}-{{{\Theta }}}_{i})}^{2})$$

where *p* is the selection strength^41^. This ensures that the maximum birth rate on *v*_*i*_ is attained for *s*_*k*_ = Θ_*i*_, which results in a differential advantage that acts as an evolutionary stabilising force. In the following we consider two habitat types denoted by **I** and **II** with symmetric environmental conditions *θ*_**I**_ and *θ*_**II**_, so that Θ_*i*_ ∈ {*θ*_**I**_, *θ*_**II**_} and *θ*_**II**_ = − *θ*_**I**_ = *θ*, where *θ* can be viewed as the habitat heterogeneity^41^.

### Deterministic approximation of the population dynamics under no selection

**Fig. 2:**
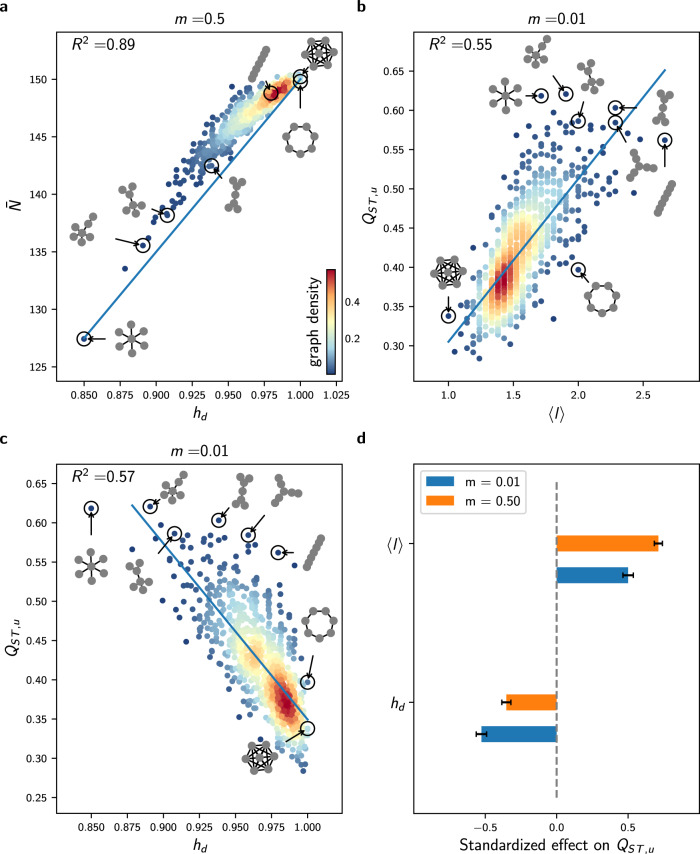
Effect of <*l*> and *h*_*d*_ on average population size $\overline{N}$ and neutral differentiation *Q*_*ST*,*u*_ in the setting with no selection. **a** Response of $\overline{N}$ to homogeneity in degree ${h}_{d}={\langle \sqrt{k}\rangle }^{2}/\langle k\rangle$ for all undirected connected graphs with *M* = 7 vertices and *m* = 0.5. **b** Response of *Q*_*ST*,*u*_ to average path length <*l*> for similar simulations obtained with *m* = 0.01. **c** Response of *Q*_*ST*,*u*_ to homogeneity in degree *h*_*d*_ for the same data. In **a**, **b**, and **c**, each dot represents average results from 5 replicate simulations of the IBM, the colour scale corresponds to the proportion of the graphs with similar *x* and *y-*axis values (graph density), and the blue line corresponds to a linear fit. **d** Standardized effect of *h*_*d*_ and <*l*> on *Q*_*ST*,*u*_, obtained from multivariate regression models independently fitted on similar data obtained for *m* = 0.01 and *m* = 0.5. The contributions of <*l*> and *h*_*d*_ to *Q*_*ST*,*u*_ are alike for low migration regimes. Error bars show 95% confidence intervals. Analogous results on graphs with *M* = 9 vertices are presented in Supplementary Fig. 1 and all regression details can be found in Supplementary Table 2.

The model can be formulated as a measure-valued point process (^30^ and Supplementary Note). Under this formalism, we demonstrate in the Supplementary Note how the population size and the trait dynamics show a deterministic behaviour when a stabilising force dampens the stochastic fluctuations. This makes it possible to express the dynamics of the macroscopic properties with deterministic differential equations, connecting emergent patterns to the processes that generate them. In particular, in the setting of no selection, competition stabilises the population size fluctuations, and the dynamics can be considered deterministic and expressed as

3 $${\partial }_{t}{N}_{t}^{(i)}={N}_{t}^{(i)}\left[b(1-m)-\frac{{N}_{t}^{(i)}}{K}\right]+mb\mathop{\sum}\limits_{j\ne i}\frac{{a}_{i,j}}{{d}_{j}}{N}_{t}^{(j)}$$

where $A={({a}_{i,j})}_{1\le i,j\le M}$ is the adjacency matrix of the graph *G* and *D* = (*d*_1_,*d*_2_,…,*d*_*M*_) is a vector containing the degree of each vertex (number of edges incident to the vertex). The first term on the right-hand side corresponds to logistic growth, which accounts for birth and death events of non-migrating individuals. The second term captures the gains due to migrations, which depend on the graph topology. Assuming that all vertices with the same degree have an equivalent position on the graph, corresponding to a “mean field” approach (see Methods), one can obtain a closed-form solution from Eq. (3) (see Eq. (12)), which shows that the average population size $\overline{N}$ scales with ${\langle \sqrt{k}\rangle }^{2}/\langle k\rangle$, where 〈*k*〉 is the average vertex degree and $\langle \sqrt{k}\rangle$ is the average square-rooted vertex degree. The quantity ${\langle \sqrt{k}\rangle }^{2}/\langle k\rangle$, denoted as *h*_*d*_, relates to the homogeneity in vertex degree of the graph and can therefore be viewed as a measure negatively associated with heterogeneity in connectivity. Simulations of the IBM illustrate that *h*_*d*_ can explain differences in population size for complex graph topologies with varying migration regimes (Fig. 2a for graphs with *M* = 7 vertices and Supplementary Fig. 1a for *M* = 9). This analytical result is connected to theoretical work on reaction-diffusion processes^49^ and highlights that irregular graphs (graphs whose vertices do not have the same degree) result in unbalanced migration fluxes that affect the ecological balance between births and deaths. Highly connected vertices present an oversaturated carrying capacity (*N*^(*i*)^ > *bK*, see Methods), increasing local competition and lowering total population size compared with regular graphs (Fig. 2a). Because populations with small sizes experience more drift (^31^ and Supplementary Fig. 2), this result indicates that graph topology affects neutral differentiation not only through population isolation, but also by affecting population dynamics.

Nonetheless, the stochasticity of the processes at the individual level can propagate to the population level and substantially affect the macroscopic properties. In particular, neutral differentiation emerges from the stochastic fluctuations of the populations’ neutral trait distribution. These fluctuations complicate an analytical underpinning of the dynamics, and in this case simulations of IBM offer a straightforward approach to evaluate the level of neutral differentiation.

### Effect of graph topology on neutral differentiation under no selection

We study a setting with no selection and investigate the effect of the graph topology on neutral differentiation. When migration is limited, individuals’ traits are coherent on each vertex but stochastic drift at the population level generates neutral differentiation between the vertices. Migration attenuates neutral differentiation because it has a correlative effect on local trait distributions. Following^21,22,26^, we expect that the intensity of the correlative effect depends on the average path length of the graph 〈*l*〉, defined as the average shortest path between all pairs of vertices^50^. For a constant number of vertices, 〈*l*〉 is strictly related to the mean betweenness centrality and quantifies the graph connectivity^50^. High 〈*l*〉 implies low connectivity and greater isolation of populations, and hence we expect that graphs with high 〈*l*〉 are associated with high differentiation levels. We consider various graphs with an identical number of vertices and run simulations of the IBM to obtain the neutral differentiation level *Q*_*ST*,*u*_ attained after a time long enough to discard transient dynamics (see Methods). We then interpret the discrepancies in *Q*_*ST*,*u*_ across the simulations by relating them to the underlying graph topologies.

We observe strong differences in *Q*_*ST*,*u*_ across graphs for varying *m*, and find that 〈*l*〉 explains at least 55% of the variation in *Q*_*ST*,*u*_ across all graphs with *M* = 7 vertices for (Fig. 2b). Nonetheless, some specific graphs, such as the star graph, present higher levels of *Q*_*ST*,*u*_ than expected by their average path length. To explain this discrepancy, we explore the effect of homogeneity in vertex degree *h*_*d*_, as we showed in Eq. (12) that it decreases population size, which should in turn increase *Q*_*ST*,*u*_ by intensifying stochastic drift. We find that *h*_*d*_ explains 57% of the variation for low *m* (Fig. 2c). However, the fit remains similar after correcting for differences in population size (see Supplementary Table 1), indicating that irregular graphs structurally amplify the isolation of populations. Unbalanced migration fluxes lead central vertices to host more individuals than allowed by their carrying capacity. This causes increased competition that results in a higher death rate, so that migrants have a lower probability of further spreading their trait. Highly connected vertices therefore behave as bottlenecks, increasing the isolation of peripheral vertices and consequently amplifying *Q*_*ST*,*u*_.

We then evaluate the concurrent effect of 〈*l*〉 and *h*_*d*_ on *Q*_*ST*,*u*_ with a multivariate regression model that we fit independently for low and high migration regimes (Fig. 2d). The multivariate regression model explains at least 70% of the variation in *Q*_*ST*,*u*_ for the migration regimes considered and for graphs with *M* = 7 vertices (see Supplementary Table 2 for details). Moreover, we find that 〈*l*〉 and *h*_*d*_ have akin contributions to neutral differentiation for low *m*, but the effect of 〈*l*〉 increases for higher migration regimes while the effect of *h*_*d*_ decreases. To ensure that these conclusions can be generalised to larger graphs, we conduct the same analysis on a subset of graphs with *M* = 9 vertices and find congruent results (Supplementary Fig. 1). In the absence of selection and with competitive interactions, graphs with a high average path length 〈*l*〉 and low homogeneity in vertex degree *h*_*d*_, or similarly graphs with low connectivity and high heterogeneity in connectivity, show high levels of neutral differentiation.

### Deterministic approximation of the population dynamics and adaptation under heterogeneous selection

We next consider heterogeneous selection and investigate the response of adaptive differentiation to the spatial distribution of habitat types, denoted as the Θ-spatial distribution. Adaptive differentiation emerges from local adaptation, but migration destabilises adaptation as a result of the influx of maladaptive migrants. We expect that higher connectivity between vertices of similar habitat type increases the level of adaptive differentiation, because it increases the proportion of well-adapted migrants. Local adaptation can be investigated by approximating the stochastic dynamics of the trait distribution with a deterministic partial differential equation (PDE). We demonstrate under mean-field assumption how the deterministic approximation can be reduced to an equivalent two-habitat model. We analyse the reduced model with the theory of adaptive dynamics^36,41^ and find a critical migration threshold *m*^⋆^ that determines local adaptation. *m*^⋆^ depends on a quantity coined the habitat assortativity *r*_Θ_, and we demonstrate with numerical simulations that *r*_Θ_ determines the overall adaptive differentiation level *Q*_*ST*,*s*_ reached at steady state in the deterministic approximation.

Heterogeneous selection, captured by the dependence of the birth rate on Θ_*i*_, generates a stabilising force that dampens the stochastic fluctuations of the adaptive trait distribution. The dynamics of the adaptive trait distribution consequently shows a deterministic behavior and we demonstrate in the Supplementary Note and Supplementary Figs. 3 and 4 that the number of individuals on *v*_*i*_ with traits $s\in {{\Omega }}\subset {{{{{{{\mathcal{S}}}}}}}}$ can be approximated by the quantity ∫_Ω_*n*^(*i*)^(*s*)*ds*, where *n*^(*i*)^ is a continuous function solution of the PDE

4 $${\partial }_{t}{n}_{t}^{(i)}(s)=	\, {n}_{t}^{(i)}(s)\left[{b}^{(i)}(s)(1-m)-\frac{1}{K}{\int}_{{{{{{{{\mathcal{S}}}}}}}}}{n}_{t}^{(i)}({{{{{{{\bf{s}}}}}}}})d{{{{{{{\bf{s}}}}}}}}\right]\\ 	 +m\mathop{\sum}\limits_{j\ne i}{b}_{j}(s)\frac{{a}_{i,j}}{{d}_{j}}{n}_{t}^{(j)}(s)+\frac{1}{2}\mu {\sigma }_{\mu }^{2}{{{\Delta }}}_{s}\left[{b}^{(i)}(s){n}_{t}^{(i)}(s)\right]$$

Equation (4) is similar to Eq. (3), except that it incorporates an additional term corresponding to mutation processes and that the birth rate is trait-dependent. We show how Eq. (4) can be reduced to an equivalent two-habitat model under mean-field assumption. The mean-field approach differs slightly from the setting with no selection because vertices are labelled with Θ_*i*_. Here we assume that vertices with similar habitat types have an equivalent position on the graph (see Supplementary Fig. 5 for a graphical representation), so that all vertices with habitat type **I** are characterised by the identical adaptive trait distribution that we denote by ${\overline{n}}^{{{{{{{{\bf{I}}}}}}}}}$, and are associated with the birth rate ${b}^{{{{{{{{\bf{I}}}}}}}}}(s)=b(1-p{(s-{\theta }_{{{{{{{{\bf{I}}}}}}}}})}^{2})$. Let *P*(**I**, **II**) denote the proportion of edges connecting a vertex *v*_*i*_ of type **II** to a vertex *v*_*j*_ of type **I**, and let *P*(**I**) denote the proportion of vertices *v*_*i*_ of type **I**. By further assuming that habitats are homogeneously distributed on the graph so that $P({{{{{{{\bf{I}}}}}}}})=P({{{{{{{\bf{II}}}}}}}})=\frac{1}{2}$, Eq. (4) transforms into

5 $${\partial }_{t}{\overline{n}}_{t}^{{{{{{{{\bf{I}}}}}}}}}(s)=	\,{\overline{n}}_{t}^{{{{{{{{\bf{I}}}}}}}}}(s)\left[{b}^{{{{{{{{\bf{I}}}}}}}}}(s)(1-m)-\frac{1}{K}{\int}_{{{{{{{{\mathcal{S}}}}}}}}}{\overline{n}}_{t}^{{{{{{{{\bf{I}}}}}}}}}({{{{{{{\bf{s}}}}}}}})d{{{{{{{\bf{s}}}}}}}}\right]+\frac{1}{2}\mu {\sigma }_{\mu }^{2}({{{\Delta }}}_{s}{b}^{{{{{{{{\bf{I}}}}}}}}}{\overline{n}}_{t}^{{{{{{{{\bf{I}}}}}}}}})(s)\\ 	 +\frac{m}{2}\,[(1-{r}_{{{\Theta }}}){b}^{{{{{{{{\bf{II}}}}}}}}}(s){\overline{n}}_{t}^{{{{{{{{\bf{II}}}}}}}}}(s)+(1+{r}_{{{\Theta }}}){b}^{{{{{{{{\bf{I}}}}}}}}}(s){\overline{n}}_{t}^{{{{{{{{\bf{I}}}}}}}}}(t)]$$

(see Methods), where we define

6 $${r}_{{{\Theta }}}=2\left(P({{{{{{{\bf{I}}}}}}}},{{{{{{{\bf{I}}}}}}}})-P({{{{{{{\bf{I}}}}}}}},{{{{{{{\bf{II}}}}}}}})\right)$$

as the habitat assortativity of the graph, which ranges from −1 to 1. When *r*_Θ_ = − 1, all edges connect dissimilar habitat types (disassortative graph), while as *r*_Θ_ tends towards 1 the graph is composed of two clusters of vertices with identical habitat types (assortative graph). Eq. (5) can be analysed with the theory of adaptive dynamics^36,38,41^, a mathematical framework that provides analytical insights by assuming a “trait substitution process”. Following this assumption, the mutation term in Eq. (5) is omitted and the phenotypic distribution results in a collection of discrete individual types that are gradually replaced by others until evolutionary stability is reached (see Methods and^36,38,41^ for details). By applying the theory of adaptive dynamics, we find a critical migration rate *m*^⋆^

7 $${m}^{\star }=\frac{1}{(1-{r}_{{{\Theta }}})}\frac{4p{\theta }^{2}}{(1+3p{\theta }^{2})}$$

so that when *m* > *m*^⋆^, a single type of individual exists with adaptive trait ${s}^{* }=\left({\theta }_{{{{{{{{\bf{II}}}}}}}}}+{\theta }_{{{{{{{{\bf{I}}}}}}}}}\right)/2=0$ in the steady-state (see Methods for the derivation of Eq. (7)). In this case, adaptive differentiation *Q*_*ST*,*s*_ is nil and the average population size is given by $\overline{N}=bK{(1-p\theta )}^{2}$. In contrast, when *m* = 0 and/or *r*_Θ_ = 1, all individuals are locally well-adapted with trait Θ_*i*_ on *v*_*i*_, and it follows that the average population size is higher and equal to $\overline{N}=bK$, while adaptive differentiation is maximal and equal to ${Q}_{ST,s}={{{{{{{\rm{Var}}}}}}}}({{\Theta }})/\left({{{{{{{\rm{Var}}}}}}}}({{\Theta }})+0\right)=1$. When 0 < *m* < *m*^⋆^, the coexistence of two types of individuals on each vertex *v*_*i*_ is predicted but the calculation of the trait values is more subtle. To understand the effect of *m* and *r*_Θ_ on the local trait distributions and on *Q*_*ST*,*s*_, we therefore leave behind the adaptive dynamics framework and numerically solve Eq. (5) by including the mutation term. When 0 < *m* < *m*^⋆^, the local trait distributions are bimodal with peaks corresponding to the two types of individuals predicted by the adaptive dynamics. The highest peak corresponds to the well-adapted individuals, whose adaptation is destabilised by the influx of maladaptive migrants (Fig. 3a). This phenomenon is dampened as *r*_Θ_ increases, since the proportion of maladaptive migrants is reduced in assortative graphs (Fig. 3b). As a consequence, the habitat assortativity *r*_Θ_ increases the differentiation *Q*_*ST*,*s*_ when 0 < *m* < *m*^⋆^ (Fig. 3c). The simulations further confirm that the adaptive dynamics prediction given by Eq. (7) is still valid when the continuous accumulation of mutations is considered, so that for *m* > *m*^⋆^ the local trait distributions obtained from Eq. (5) are unimodal and *Q*_*ST*,*s*_ vanishes (Fig. 3a,c). Our analysis of the mean-field deterministic approximation Eq. (5) therefore demonstrates that assortative graphs present high levels of adaptive differentiation *Q*_*ST*,*s*_. On the other hand, the analysis shows that *Q*_*ST*,*s*_ rapidly declines with increasing *m* on disassortative graphs, until *Q*_*ST*,*s*_ vanishes when *m* > *m*^⋆^.

**Fig. 3:**
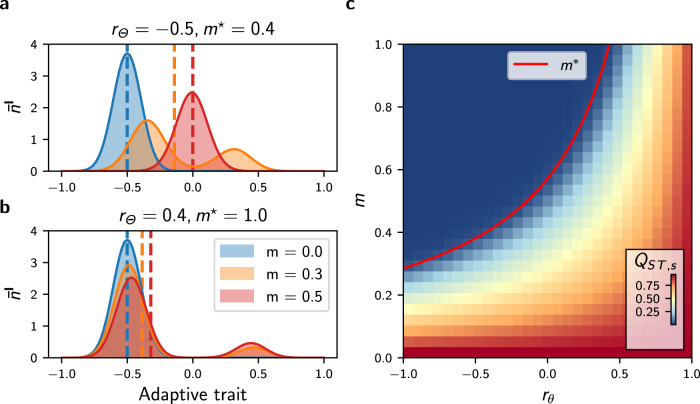
Effect of habitat assortativity *r*_Θ_ and migration *m* on the local adaptive trait distribution ${\overline{n}}^{{{{{{{{\bf{I}}}}}}}}}$ and on the adaptive differentiation level *Q*_*ST*,*s*_ under the mean field, deterministic approximation Eq. (5). **a** Effect of *m* and *r*_Θ_ on ${\overline{n}}^{{{{{{{{\bf{I}}}}}}}}}$. Migration induces the apparition of maladaptive individuals (centred around *θ*_**II**_ = 0.5), which destabilise local adaptation by displacing the mean value of the well-adapted individuals (centred around *θ*_**I**_ = − 0.5). Together with the decrease in local adaptation, migration causes a displacement of the mean value of the local trait distribution (represented by the vertical dashed lines), which decreases local population size and adaptive differentiation *Q*_*ST*,*s*_. **b** Similar data for higher *r*_Θ_. Increasing *r*_Θ_ increases population size and *Q*_*ST*,*s*_. **c** Effect of *r*_Θ_ on *Q*_*ST*,*s*_. The red line indicates the critical migration threshold *m*^⋆^ predicted by Eq. (7); *Q*_*ST*,*s*_ vanishes when *m* > *m*^⋆^.

### Effect of graph topology on adaptive differentiation under heterogeneous selection

**Fig. 4:**
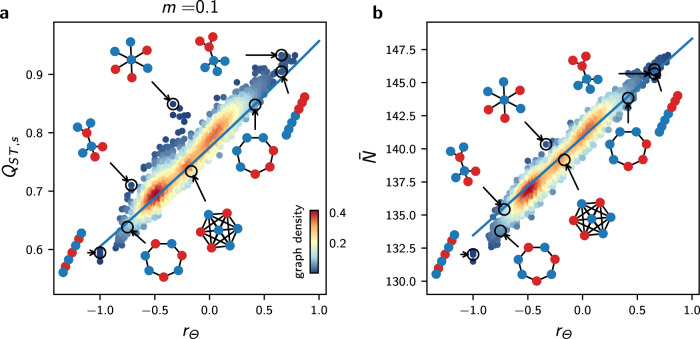
Effect of habitat heterogeneity *r*_Θ_ on *Q*_*ST*,*s*_ and average population size $\overline{N}$ for general graph ensembles. **a** Effect of *r*_Θ_ on *Q*_*ST*,*s*_ for all undirected connected graphs with *M* = 7 vertices and varying *r*_Θ_, for *m* = 0.1. **b** Effect of *r*_Θ_ on average population size $\overline{N}$ for the same simulations. In **a** and **b**, each dot represents average results from 5 replicate simulations of the IBM, the colour scale corresponds to the proportion of the graphs with similar *x* and *y* axis values (graph density), and the blue lines correspond to results obtained from the mean-field approximation Eq. (5). Insights from Eq. (5) are congruent with the IBM simulations for complex habitat connectivity patterns at low *m*. Similar results with *m* = 0.5 are presented in Supplementary Fig. 6.

**Fig. 5:**
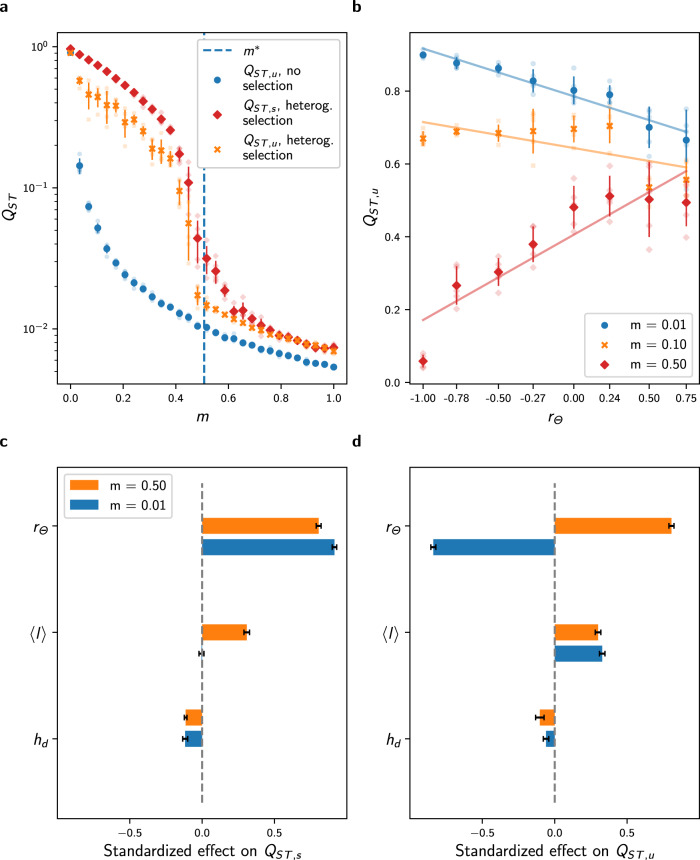
Effect of *r*_Θ_, 〈*l*〉, and *h*_*d*_ on *Q*_*ST*,*s*_ and *Q*_*ST*,*u*_ in the setting with heterogeneous selection. **a** Comparison of the response of *Q*_*ST*,*u*_ to migration with the response of *Q*_*ST*,*u*_ in the setting with no selection for the complete graph. The dashed vertical blue line corresponds to the critical migration regime *m*^⋆^ predicted by Eq. (7). Heterogeneous selection increases *Q*_*ST*,*u*_ when *m* < *m*^⋆^, but local adaptation is lost when *m* > *m*^⋆^, and in this case *Q*_*ST*,*u*_ reaches similar levels as *Q*_*ST*,*u*_ in the setting with no selection. **b** Response of *Q*_*ST*,*u*_ to *r*_Θ_ and migration for the path graph. *r*_Θ_ correlates positively with *Q*_*ST*,*u*_ for high *m*, but correlates negatively for low *m*. In **a**, **b**, each plain dot represents average results from 5 replicate simulations, the bars represent one standard deviation, and each fade dot represents a single replicate value. **c**, **d** Standardized effect of *h*_*d*_, 〈*l*〉, and *r*_Θ_ on *Q*_*ST*,*s*_, and *Q*_*ST*,*u*_ obtained from a multivariate regression model independently fitted for low and high migration regimes on average results from 5 replicate simulations of the IBM on all undirected connected graphs with *M* = 7 vertices and varying *r*_Θ_ (see Methods). The ambivalence of the effect of *r*_Θ_ on *Q*_*ST*,*u*_ found for the path graph holds for general graph ensembles and adds up to that of 〈*l*〉 and *h*_*d*_. Error bars show 95% confidence intervals. Analogous results on graphs with *M* = 9 vertices are presented in Supplementary Fig. 7 and all regression details can be found in Supplementary Table 3.

To generalise the conclusions drawn from the mean-field deterministic approximation Eq. (5), we generate different Θ-spatial distributions for varying graph topology, and compare outputs of the IBM simulations with those of Eq. (5) (see Methods for the details of the simulations). For each combination of Θ-spatial distribution and graph, we compute the habitat assortativity *r*_Θ_, since *r*_Θ_ can be generalised from Eq. (6) to any graph topology following the original definition of^51^ as

8 $${r}_{{{\Theta }}}=\frac{{{{{{{{\rm{Cov}}}}}}}}({{{\Theta }}}_{\times },{{{\Theta }}}_{\wedge })}{{\sigma }_{{{{\Theta }}}_{\times }}{\sigma }_{{{{\Theta }}}_{\wedge }}}$$

where Θ_×_ and Θ_∧_ denote the sets of habitats found at the toe and tip of each directed vertex of graph *V*, and 〈Θ_×_〉, 〈Θ_∧_〉 and ${\sigma }_{{{{\Theta }}}_{\times }},{\sigma }_{{{{\Theta }}}_{\wedge }}$ denote their respective means and standard deviations (see Supplementary Note). The mean-field deterministic approximation Eq. (5) is in very good agreement with the IBM simulations for general graph ensembles at low migration regimes, and captures the response of $\overline{N}$ and *Q*_*ST*,*s*_ to *r*_Θ_ (Fig. 4). Nonetheless, under high migration regimes, higher levels of *Q*_*ST*,*s*_ are observed in the stochastic simulations compared with the mean field deterministic approximation (Supplementary Fig. 6). We hypothesize that this reinforcement is generated by stochastic drift, which must become the main driver of differentiation when local adaptation is lost for *m* > *m*^⋆^, and perform a multivariate regression analysis to investigate the additional effect of 〈*l*〉 and *h*_*d*_ on *Q*_*ST*,*s*_. As expected, the analysis highlights that the effect of 〈*l*〉 and *h*_*d*_ are substantial and complement the effect of *r*_*θ*_ for high *m* (Fig. 5c for graphs with *M* = 7 vertices and Supplementary Fig. 7a for *M* = 9), further explaining the discrepancies observed (see Supplementary Table 3).

We extend our analyses to realistic landscapes with a continuum of habitat types by running simulations on graphs obtained from real spatial habitat datasets and by considering mean annual temperature as a proxy for habitat type (see Supplementary Fig. 8 and Supplementary Table 4). We also consider simulations accounting for trait-dependent competition to test whether our results hold under more complex ecological processes (see Supplementary Note for the implementation details and Supplementary Table 5 for the results). The simulations are congruent and show that the effects of *r*_Θ_, *h*_*d*_, and 〈*l*〉 are similar under these alternative settings, underlining the robustness of these metrics and the generality of our conclusions. Taken together, these results indicate that under sufficiently strong selection and sufficiently high habitat heterogeneity, adaptive differentiation *Q*_*ST*,*s*_ is mainly driven by habitat assortativity *r*_Θ_. Nonetheless, local adaptation is lost in disassortative graphs when *m* > *m⋆*, such that 〈*l*〉 and *h*_*d*_ become complementary determinants of *Q*_*ST*,*s*_ for high migration regimes.

### Effect of habitat assortativity on neutral differentiation under heterogeneous selection

We finally consider a setting with heterogeneous selection where individuals carry both neutral and adaptive traits. With distinct habitat types, selection promotes neutral differentiation by reducing the birth rate of maladaptive migrants, reinforcing the isolation of local populations. We have shown above that adaptive differentiation *Q*_*ST*,*s*_ is driven by habitat assortativity *r*_Θ_, so we expect *r*_Θ_, together with the topological metrics found in the setting with no selection, to influence the level of neutral differentiation *Q*_*ST*,*u*_. We first investigate how the response of *Q*_*ST*,*u*_ to migration compares between the setting with no selection and the setting with heterogeneous selection for graphs with an identical topology. We then examine how the response compares between graphs with an identical topology but different *r*_Θ_. We finally consider simulations on different graphs with varying *r*_Θ_ to assess the concurrent effect of 〈*l*〉, *h*_*d*_, and *r*_Θ_ on *Q*_*ST*,*u*_.

Migration has a fitness cost because maladaptive migrants present lower fitness. Under an equivalent migration regime, migrants therefore have a lower probability of reproduction, increasing the populations’ isolation compared with a setting without selection. Simulations with varying *m* on the complete graph confirm that selection in heterogeneous habitats reinforces *Q*_*ST*,*u*_ compared with a setting without selection (Fig. 5a). Nonetheless, previous results show that adaptive differentiation *Q*_*ST*,*s*_ vanishes on a disassortative graph when *m* > *m*^⋆^, implying that individuals become equally fit in all habitats. In this case, the isolation effect of heterogeneous selection is lost and *Q*_*ST*,*u*_ reaches a similar level as in the setting with no selection for *m* > *m*^⋆^ (Fig. 5a), although *Q*_*ST*,*u*_ is slightly higher in the setting with heterogeneous selection due to lower population size ($\overline{N}=bK(1-p\theta )$ vs. $\overline{N}=bK$, see section above and Methods). This suggests that *r*_Θ_ reinforces *Q*_*ST*,*u*_, as assortative graphs sustain higher levels of adaptive differentiation (Figs. 3 and 4). Simulations on the path graph with varying Θ-spatial distribution support this conclusion for high migration regimes, but show the opposite relationship under low migration regimes, where the habitat assortativity *r*_Θ_ decreases *Q*_*ST*,*u*_ (Fig. 5b). Assortative graphs are composed of large clusters of vertices with similar habitats, within which migrants can circulate without fitness losses. Local neutral trait distributions become more correlated within these clusters, resulting in a decline in *Q*_*ST*,*u*_ for assortative graphs compared with disassortative graphs. Figure 5b therefore highlights the ambivalent effect of *r*_Θ_ on *Q*_*ST*,*u*_. *r*_Θ_ reinforces *Q*_*ST*,*u*_ by favouring adaptive differentiation, but also decreases *Q*_*ST*,*u*_ by decreasing population isolation within clusters of vertices with the same habitat type.

We compare the effect of *r*_Θ_ on *Q*_*ST*,*u*_ to the effect of the topology metrics 〈*l*〉 and *h*_*d*_ found in the setting with no selection using multivariate regression analysis on simulation results obtained for different graphs with varying Θ-spatial distribution (Fig. 5d for graphs with *M* = 7 vertices and Supplementary Fig. 7b for *M* = 9). The multivariate model explains the discrepancies in *Q*_*ST*,*u*_ across the simulations for low and high migration regimes (see Supplementary Table 3 for details), and we find that *r*_Θ_, 〈*l*〉, and *h*_*d*_ contribute similarly to neutral differentiation. Hence, the effects of *r*_Θ_ and the topology metrics 〈*l*〉 and *h*_*d*_ add up under heterogeneous selection. A change in sign of the standardized effect of *r*_Θ_ on *Q*_*ST*,*s*_ for low and high migration regimes verifies that the ambivalent effect of *r*_Θ_ on *Q*_*ST*,*u*_ found on the path graph holds for general graph ensembles. Simulations with trait-dependent competition and simulations on realistic graphs with a continuum of habitat types equally confirm the ambivalent effect of *r*_Θ_ and further support the complementary effect of 〈*l*〉 and *h*_*d*_ on *Q*_*ST*,*u*_ (see Supplementary Fig. 8). 〈*l*〉 and *h*_*d*_ therefore drive neutral differentiation with and without heterogeneous selection. *r*_Θ_ becomes an additional determinant of neutral differentiation under heterogeneous selection. In contrast to the non-ambivalent, positive effect of habitat assortativity on adaptive differentiation, *r*_Θ_ can amplify or depress neutral differentiation depending on the migration regime considered.

## Discussion

Using analytical tools and simulations, we have built upon a graph representation of landscapes and a stochastic individual-based model to investigate how landscape features drive phenotypic differentiation. Our study is based on a first-principles modelling approach^29^ describing the stochastic dynamics of individuals and capturing the interplay between population dynamics, phenotypic evolution, and spatial dynamics in heterogeneous habitats. In contrast to metacommunity models^17–23^ and evolutionary metacommunity models^26,27^, we have focused on differentiation at the population level. Quantitative genetics and population genetics studies have investigated the effect of topology on differentiation under the assumption of non-overlapping generations, constant population sizes, and regular spatial structures^31,33,34,48,52^. Generalising beyond these assumptions, our modelling framework accounts for population dynamics and includes competition and frequency-dependent selection. The systematic investigation of the effect of topology on differentiation over general graph ensembles and under different ecological settings shows that average path length 〈*l*〉, homogeneity in vertex degree *h*_*d*_, and habitat assortativity *r*_Θ_ contribute equally to differentiation. These results support correlative studies that have associated population differentiation^44,53^ and species richness^4,5,54–59^ with a variety of metrics used as surrogates for connectivity, connectivity heterogeneity, and habitat heterogeneity. To further our understanding of the origin of spatial biodiversity patterns, the contribution of landscape properties to discrepancies in population differentiation could be investigated at large scales by (i) using techniques to project real landscapes on graphs (see Supplementary Fig. 8a, b); (ii) characterising the landscape features with 〈*l*〉, *h*_*d*_ and *r*_Θ_; and (iii) relating the obtained metrics maps to observation data. More generally, the proposed eco-evolutionary model on spatial graphs could be combined with inference methods to estimate ecological, spatial, and evolutionary processes of real populations from observation data, similarly to^60^. This approach might improve current inferential techniques based on models that do not account for competition nor heterogeneous selection (see e.g.^61^). Overall, our results point to topology metrics that can connect spatial biodiversity patterns to the generating eco-evolutionary and spatial processes.

In the absence of selection, neutral differentiation is more pronounced on graphs with a high average path length 〈*l*〉, but is also negatively associated with homogeneity in degree *h*_*d*_ (Fig. 2c, d). 〈*l*〉 generalises the concept of dimensionality in^33,34,48^, where it is shown that differentiation is lower for two-dimensional grid graphs compared with path graphs. 〈*l*〉 also closely relates to the concept of resistance distance shown theoretically and empirically to drive genetic differentiation^53,62^. At the species level, a similar effect of 〈*l*〉 on *β*-diversity (pairwise differences in species composition) has been reported with the graph metacommunity model of^21^ and with the graph eco-evolutionary metacommunity model of^26^. Accounting for population dynamics and specifically including competition processes, we have shown that not only 〈*l*〉 but also *h*_*d*_ affects neutral phenotypic differentiation (Fig. 2c, d). Our model realistically assumes that population growth is limited by the local carrying capacity. The latter becomes saturated on highly connected vertices in irregular graphs, an effect that has been experimentally documented in microcosm experiments^63^. As a consequence, central vertices behave as bottlenecks and amplify the isolation of peripheral vertices^13^. The role of *h*_*d*_ cannot be captured with classical metapopulation and quantitative genetics models or with models of evolutionary dynamics in graphs, as they assume constant population size. This behaviour should be prevalent in patchy landscapes where interspecific competition is high because of limiting resources. Our study highlights that heterogeneity in connectivity can reinforce differentiation patterns through the creation of unbalanced migration fluxes which affect ecological equilibrium.

Habitat assortativity *r*_Θ_ is a useful indicator for assessing how the spatial distribution of habitat types modulates local adaptation and adaptive differentiation in complex landscapes^64^. While adaptation has been extensively studied along environmental gradients^32,35,40,65–68^, landscapes can be patchy and it is unrealistic to assume regularity^16^. Our model of heterogeneous selection on spatial graphs extends the two-habitat setting investigated in^36,38,41,52^ and captures irregularity in connectivity between distinct habitats^16^. Similarly to the aforementioned studies, we have found a critical migration regime *m*^⋆^ that dictates the possibility of adaptation. Equation (7) indicates that *m*^⋆^ increases with increasing selection strength *p* and with increasing environmental heterogeneity *θ*, the latter playing a similar role as the slope of the environmental gradient in^32,40,65,67^. Local adaptation would consequently be sustained under higher migration regimes following an increase in these parameters. Additionally, the critical migration regime *m*^⋆^ in Eq. (7) involves the habitat assortativity *r*_Θ_, which must be regarded as a measure of habitat spatial auto-correlation based on the dispersal range of a species^64^. Our results indicate that for general habitat distributions, *r*_Θ_ is the main determinant of adaptive differentiation under sufficiently strong selection *p* and high habitat heterogeneity *θ*, irrespective of the graph topology (Fig. 5c, Supplementary Fig. 7a, and Supplementary Fig. 8). As *p* decreases, however, the effect of stochastic drift on *Q*_*ST*,*s*_ should increase, and in this case, the topology metrics 〈*l*〉 and *h*_*d*_ should become the most important determinants of *Q*_*ST*,*s*_. Our results predict that in landscapes with heterogeneous habitats and where selection is strong, populations structured over assortative habitats are larger, support higher adaptive differentiation, and can be locally well-adapted even in the case where migration rates are high.

Spatial eco-evolutionary feedbacks in heterogeneous habitats can critically affect differentiation^64^. While most eco-evolutionary studies have investigated diversification by considering a unique adaptive trait^35,40,66,67^, distinguishing between neutral and adaptive processes is crucial^9^ and our work underlines the distinct responses of neutral and adaptive differentiation to landscape features (Fig. 5c vs. d). Our study builds upon recent mathematical models that consider the co-evolution of neutral and adaptive traits^42,43^ and extends those works to a spatial context. Our work provides an analytical framework to the concept of isolation by environment (IBE)^13^, which has been suggested to be one of the most important mechanisms governing differentiation in nature^14^. Heterogeneous selection leads to more isolation by modifying the fitness of migrants^40^, which further reduces gene flow^64^ and therefore affects the level of neutral differentiation (Fig. 5a)^15^. Our work proposes a mechanism by which habitat assortativity, relative to the migration regime, controls the direction of the effect of habitat heterogeneity on differentiation (Fig. 5d). Patchy, heterogeneous habitats can promote neutral differentiation as a result of selection that reduces effective migration^59^. Nonetheless, adaptive differentiation decreases substantially when migration is high relative to the critical migration regime *m*^⋆^. In this case, neutral differentiation should be higher in landscapes with more aggregated habitats^64^. Our study suggests that habitat assortativity must be considered for a complete understanding of differentiation in complex environments^59^.

In conclusion, we have established how differentiation can emerge at the population level from eco-evolutionary feedbacks in complex landscapes by using an analytical description of micro-evolutionary processes explicitly accounting for spatial dynamics over graphs. Our study formalises how differentiation emerges from the interplay between spatial dynamics, the co-evolution of neutral and adaptive traits, and landscape properties. Connectivity and habitat assortativity emerge as core determinants of differentiation in spatial graphs. These results resonate with empirical findings and previous theoretical works. Our study further stresses that habitat assortativity can depress or foster neutral differentiation depending on the migration regime. Additionally, our work highlights that heterogeneity in connectivity is an equally strong determinant of differentiation because highly connected habitats behave as bottlenecks, increasing the isolation of peripheral habitats. The present approach offers a promising framework for studying complex adaptive systems, as it can elucidate how macroscopic properties emerge from microscopic processes acting upon agents structured over complex spatio-evolutionary structures.

## Methods

### Mean-field approximation

In the setting with no selection, the mean-field approach involves the assumption that all vertices having the same degree are equivalent. For this, let $P(k,k^{\prime} )$ denote the proportion of edges that map a vertex with degree *k* to a vertex with degree $k^{\prime}$, and consider the average population size ${\overline{N}}_{t}^{(k)}$ in each vertex with degree *k* at time *t*. An individual has probability $P(k,k^{\prime} )/k^{\prime}$ to migrate from a vertex with degree $k^{\prime}$ to a vertex with degree *k*. Viewing *a*_*i*,*j*_/*d*_*j*_ as the probability that an individual on *v*_*i*_ chosen for migration moves to *v*_*j*_, Eq. (3) then transforms into

9 $${\partial }_{t}{\overline{N}}_{t}^{(k)}={\overline{N}}_{t}^{(k)}\left[b(1-m)-\frac{{\overline{N}}_{t}^{(k)}}{K}\right]+mbk\mathop{\sum}\limits_{k^{\prime} \in V}\frac{P(k,k^{\prime} )}{k^{\prime} }{\overline{N}}_{t}^{(k^{\prime} )}$$

Assuming uncorrelated graphs for which $P(k,k^{\prime} )/k^{\prime} =P(k^{\prime} )k^{\prime} /\langle k\rangle$, where 〈*k*〉 denotes the average degree of the graph^49^, yields

10 $${\partial }_{t}{\overline{N}}_{t}^{(k)}={\overline{N}}_{t}^{(k)}\left[b(1-m)-\frac{{\overline{N}}_{t}^{(k)}}{K}\right]+mb\frac{k}{\langle k\rangle }{\overline{N}}_{t}$$

where

11 $${\overline{N}}_{t}=\mathop{\sum}\limits_{k}P(k){\overline{N}}_{t}^{(k)}.$$

When solving for the stationary state and setting *m* = 1, one obtains ${\overline{N}}^{(k)}=\sqrt{bK\frac{k}{\langle k\rangle }\overline{N}}$ from Eq. (10). Combining this with Eq. (11) yields

12 $$\overline{N}=bK{\langle \sqrt{k}\rangle }^{2}/\langle k\rangle$$

In the setting with heterogeneous selection, the mean-field approach involves the assumption that all vertices with a similar habitat are equivalent. In this case, an individual from a vertex of habitat type **II** has the probability *P*(**I**, **II**)/*P*(**II**) of migrating to a vertex of type **I**, and therefore Eq. (4) transforms into

13 $${\partial }_{t}{\overline{n}}_{t}^{{{{{{{{\bf{I}}}}}}}}}(s)=	\,{\overline{n}}_{t}^{{{{{{{{\bf{I}}}}}}}}}(s)\left[{b}^{{{{{{{{\bf{I}}}}}}}}}(s)(1-m)-\frac{1}{K}{\int}_{{{{{{{{\mathcal{S}}}}}}}}}{\overline{n}}_{t}^{{{{{{{{\bf{I}}}}}}}}}({{{{{{{\bf{s}}}}}}}})d{{{{{{{\bf{s}}}}}}}}\right]+\frac{1}{2}\mu {\sigma }_{\mu }^{2}{{{\Delta }}}_{s}\left[{b}^{{{{{{{{\bf{I}}}}}}}}}(s){\overline{n}}_{t}^{{{{{{{{\bf{I}}}}}}}}}(s)\right]\\ 	 +m\mathop{\sum}\limits_{i\in \{{{{{{{{\bf{I}}}}}}}},{{{{{{{\bf{II}}}}}}}}\}}{b}_{i}(s)\frac{P({{{{{{{\bf{I}}}}}}}},i)}{P(i)}\,{\overline{n}}_{t}^{i}(s)$$

Considering that $P({{{{{{{\bf{II}}}}}}}})=P({{{{{{{\bf{I}}}}}}}})=\frac{1}{2}$ (habitats are equally distributed), *P*(**I**, **I**) + *P*(**I**, **II**) = *P*(**I**) (sum of conditional expectations), and ${r}_{{{\Theta }}}=2\left(P({{{{{{{\bf{I}}}}}}}},{{{{{{{\bf{I}}}}}}}})-P({{{{{{{\bf{I}}}}}}}},{{{{{{{\bf{II}}}}}}}})\right)$ (Eq. (6)), one obtains

14 $$P({{{{{{{\bf{I}}}}}}}},{{{{{{{\bf{II}}}}}}}})=\frac{1}{4}(1-{r}_{{{\Theta }}})\quad \,{{\mbox{and}}}\,\quad P({{{{{{{\bf{I}}}}}}}},{{{{{{{\bf{I}}}}}}}})=\frac{1}{4}(1+{r}_{{{\Theta }}})$$

Combining Eq. (14) with Eq. (13) yields Eq. (5). We show in the Supplementary Note how one can derive Eq. (6) from the general definition of assortativity given in Eq. (8) and initially introduced in^51^.

### Adaptive dynamics on graphs

The adaptive dynamics theory considers a monomorphic population that evolves following a “trait substitution process”^36^. Accordingly, the trait *s* of the monomorphic metapopulation evolves gradually along the direction given by its fitness gradient, until it reaches a singular strategy s* for which the fitness gradient vanishes. By omitting the mutation term, Eq. (6) can be written in the matrix form

15 $${\partial }_{t}{\overline{{{{{{{{\bf{n}}}}}}}}}}_{t}(s)=M(s,{\overline{{{{{{{{\bf{N}}}}}}}}}}_{t})\,{\overline{{{{{{{{\bf{n}}}}}}}}}}_{t}(s)$$

where ${\overline{{{{{{{{\bf{n}}}}}}}}}}_{t}=({\overline{n}}_{t}^{{{{{{{{\bf{I}}}}}}}}},{\overline{n}}_{t}^{{{{{{{{\bf{II}}}}}}}}})$ and ${\overline{{{{{{{{\bf{N}}}}}}}}}}_{t}=({\overline{N}}_{t}^{{{{{{{{\bf{I}}}}}}}}},{\overline{N}}_{t}^{{{{{{{{\bf{II}}}}}}}}})$ are the vectors containing the population densities and the population size on each habitat type, and

16 $$M(s,\overline{{{{{{{{\bf{N}}}}}}}}})=\left[\begin{array}{cc}{{\mathfrak{r}}}^{{{{{{{{\bf{I}}}}}}}}}(s,{\overline{N}}^{{{{{{{{\bf{I}}}}}}}}})&\frac{m}{2}(1-{r}_{{{\Theta }}}){b}^{{{{{{{{\bf{II}}}}}}}}}(s)\\ \frac{m}{2}(1-{r}_{{{\Theta }}}){b}^{{{{{{{{\bf{I}}}}}}}}}(s)&{{\mathfrak{r}}}^{{{{{{{{\bf{II}}}}}}}}}(s,{\overline{N}}^{{{{{{{{\bf{II}}}}}}}}})\end{array}\right]$$

is the so-called projection matrix^36^, with ${{\mathfrak{r}}}^{{{{{{{{\bf{I}}}}}}}}}(s,{\overline{N}}^{{{{{{{{\bf{I}}}}}}}}})={b}^{{{{{{{{\bf{I}}}}}}}}}(s)(1+\frac{m}{2}({r}_{{{\Theta }}}-1))-{\overline{N}}^{{{{{{{{\bf{I}}}}}}}}}/K$. The overall fitness of individuals with trait *s* is the leading eigenvalue of *M*, which we denote with $\lambda (s,\overline{{{{{{{{\bf{N}}}}}}}}})$. We obtain the singular strategy s* by setting the fitness gradient $\frac{\partial \lambda }{\partial s}(s,\overline{{{{{{{{\bf{N}}}}}}}}})=0$, from which we further obtain the demographic equilibrium ${\overline{{{{{{{{\bf{N}}}}}}}}}}^{{s}^{* }}$. Because of symmetries, we must have ${\overline{N}}^{{{{{{{{\bf{I}}}}}}}},{s}^{* }}={\overline{N}}^{{{{{{{{\bf{II}}}}}}}},{s}^{* }}$ and ${s}^{* }=\frac{{\theta }_{{{{{{{{\bf{I}}}}}}}}}+{\theta }_{{{{{{{{\bf{II}}}}}}}}}}{2}=0$, such that ${\overline{N}}^{{{{{{{{\bf{I}}}}}}}},{s}^{* }}={\overline{N}}^{{{{{{{{\bf{II}}}}}}}},{s}^{* }}=bK(1-p{\theta }^{2})$. s* is said to be evolutionary stable if no mutants can invade, i.e., if s* locally maximises the fitness of a mutant with trait *y* in the resident population with trait s*, given by $\lambda (y,{\overline{{{{{{{{\bf{N}}}}}}}}}}^{{s}^{* }})$ (see^36^ for details). One can show that ${\left[\frac{\partial \lambda }{\partial y}(y,{\overline{{{{{{{{\bf{N}}}}}}}}}}^{{s}^{* }})\right]}_{y = {s}^{* }}=0$ and the condition for evolutionary stability becomes ${\left[\frac{{\partial }^{2}\lambda }{\partial {y}^{2}}(y,{\overline{{{{{{{{\bf{N}}}}}}}}}}^{{s}^{* }})\right]}_{y = {s}^{* }} < 0$. We compute and simplify this inequality through computer algebra (see Mathematica notebook provided in the simulation code), which leads to Eq. (7).

### Numerical simulations

The model was implemented in a multi-purpose Julia package called **EvoId.jl**, available at https://github.com/vboussange/EvoId.jl. For each result presented, *b* = 1, local carrying capacity *K* = 150, selection strength *p* = 1, mutation rate *μ* = 0.1, mutation range *σ*_*μ*_ = 5⋅10^−2^, and total time span *t* = 1000. This parameter choice made it possible to discard transient dynamics while obtaining results in a reasonable computational time (see Supplementary Fig. 9). For both the setting with no selection and the setting with heterogeneous selection, we ran simulations on all of the 853 undirected connected graphs with *M* = 7 vertices and on 1126 of the 261,080 undirected connected graphs with *M* = 9 vertices, listed at http://oeis.org/A001349. Graphs with *M* = 9 vertices were selected with a stratified sampling method: we randomly sampled without replacement a maximum of 50 graphs for each class of graphs with an equal number of vertices. For the setting with heterogeneous selection, we generated the labeled graphs by randomly generating Θ-spatial distributions, and by using a stratified sampling strategy to select without replacement at most 3 and 2 Θ-spatial distributions corresponding to the quartiles of the *r*_*θ*_ values obtained, respectively for graphs with *M* = 7 and *M* = 9 vertices. This sampling strategy allowed to obtain a uniform distribution of the topology metrics investigated in the study, and therefore permitted to correctly represent the population of graphs to investigate their effect on differentiation. We then computed *Q*_*ST*,*u*_ and *Q*_*ST*,*s*_, which we further averaged over the last time steps and across the replicates. Since the dynamics of *Q*_*ST*,*u*_ is characterised by large quadratic variations, we simulated individuals with *d* = 300 neutral traits, where each trait can independently be affected by mutations. *Q*_*ST*,*u*_ values presented were then obtained from the average *Q*_*ST*,*u*_ for each trait. This reduced the variance of the numerical simulations and is also biologically meaningful because populations are characterised by many traits, most of which are neutral^9^. As initial conditions, *MK* individuals were homogeneously distributed over all of the vertices, with traits centred on 0 and with standard deviation *σ*_*μ*_. Graph metrics used for the meta-analysis were calculated using the **LightGraphs.jl** library^69^. We numerically solved the PDEs with a finite difference scheme using **DifferentialEquations.jl**^70^, ensuring that the domain was large enough to avoid border effects.

### Statistics and reproducibility

Statistical anyalses were conducted in Julia using **StatsKit.jl**. All simulations can be exactly reproduced from the code available at https://github.com/vboussange/differentiation-in-spatial-graphs.

### Reporting summary

Further information on research design is available in the Nature Research Reporting Summary linked to this article.

## Supplementary information

Supplementary Information

Reporting Summary

## Data Availability

The data underlying our figures is available at https://github.com/vboussange/differentiation-in-spatial-graphs.

## Code availability

The simulation code is available at https://github.com/vboussange/differentiation-in-spatial-graphs.
