# Supplementary material for: Eco-evolutionary model on spatial graphs reveals how habitat structure affects phenotypic differentiation
Source: Commun Biol. 2022 Jul 6;5:668. doi: 10.1038/s42003-022-03595-3 (PMC9259634; doi:10.1038/s42003-022-03595-3)
Supplement: Supplementary file 1 — Supplementary Information [file 42003_2022_3595_MOESM1_ESM.pdf]

Supplementary Material: Eco-evolutionary model on spatial graphs reveals how habitat structure affects phenotypic differentiation

Victor Boussange[a,b,1] and Loïc Pellissier[a,b,2]

[a]Swiss Federal Research Institute WSL, CH-8903 Birmensdorf, Switzerland
[b]Landscape Ecology, Institute of Terrestrial Ecosystems, Department of Environmental System Science, ETH Zürich, CH-8092 Zürich, Switzerland
[1]Email: vic.boussange@gmail.com
[2]Email: loic.pellissier@usys.ethz.ch

June 11, 2022

Contents

A Supplementary Note **1**
A.1 Mathematical construction of the model 1
A.2 Deterministic approximation 2
A.2.1 Setting with no selection 2
A.2.2 Setting with heterogeneous selection 3
A.3 Trait-dependent competition 4
A.4 Derivation of the habitat assortativity metric r_Θ in binary environments 4

B Supplementary Figures **7**

C Supplementary Tables **19**

A Supplementary Note

A.1 Mathematical construction of the model

The model is a measure-valued point process [1], so that individuals are represented as dirac functions $\delta_{x_k^{(i)}}$, where $x_k^{(i)} \in \mathcal{X}$ corresponds to the traits' value of individual k located on vertex v_i. Under this formalism, the population on v_i is represented as a sum of dirac functions $\nu^{(i)} = \sum_k^{N^{(i)}} \delta_{x_k^{(i)}}$, where $N^{(i)}$ is the local population size. It follows that the time variation of the process can be described by the so-called infinitesimal generator L, defined for all real valued functions ϕ as

$$L\phi(\nu_t^{(i)}) = \partial_t \mathbb{E}\left[\phi(\nu_t^{(i)})\right] \tag{S1}$$

(see [2] for an introduction to infinitesimal generators). Equation (S1) provides the expected time variation at time t of e.g. the population size by choosing $\phi(\nu_t^{(i)}) = \int_{\mathcal{X}} \nu_t^{(i)}(dx)$. Recall that we use $b^{(i)}$ to denote the birth rate on vertex v_i, d for the death rate, μ for the mutation probability, m for the migration probability, $\mathcal{M}(x,y) = \frac{1}{\sqrt{2\pi\sigma_\mu}} \exp\left(\frac{||x-y||^2}{2\sigma_\mu}\right)$ for the mutation kernel, K for the local carrying capacity, $A = (a_{i,j})_{1\leq i,j \leq M}$ for the adjacency matrix of the graph G, and $D = (d_1, d_2, \dots, d_M)$ for the vector containing the degree of each vertex. In order to explicitly write the generator L, let us recall that five events of different natures can alter the number of individuals with trait x on vertex v_i:

- an individual on v_i with trait x can give birth to an offspring that does not experience mutations nor migration, at rate $(1-\mu)(1-m)b^{(i)}(x)$,
- an individual on v_i with trait y can give birth to an offspring with mutated trait x that does not experience migration, at rate $\mu(1-m)\mathcal{M}(x,y)b^{(i)}(y)$,
- an individual on v_i with trait x can die, at rate $d(N^{(i)}) = \frac{N^{(i)}}{K} = \frac{1}{K}\int_{\mathcal{X}} \nu_t^{(i)}(dx)$,
- an individual on v_j with trait x can give birth to an offspring that does not experience mutations and migrates to v_i, at rate $\frac{a_{i,j}}{d_j}(1-\mu)m\, b^{(j)}(x)$,
- an individual on v_j with trait y can give birth to an offspring with mutated trait x that migrates to v_i, at rate $\frac{a_{i,j}}{d_j}\mu m \mathcal{M}(x,y) b^{(j)}(x)$.

Summing over all all individuals and all vertices yields

$$
\begin{aligned}
L\phi(\nu_t^{(i)}) = &\int_{\mathcal{X}} \left\{ b^{(i)}(\mathbf{x})(1-\mu)(1-m)(\phi(\nu_t^{(i)} + \delta_{\mathbf{x}}) - \phi(\nu_t^{(i)})) \right\} \nu_t^{(i)}(d\mathbf{x}) && \text{births w/o mutations, w/o migrations} \\
&+ \int_{\mathcal{X}} \left\{ \mu(1-m) \int_{\mathcal{X}} b^{(i)}(y)(\phi(\nu_t^{(i)} + \delta_z) - \phi(\nu_t^{(i)}))\mathcal{M}(\mathbf{x}, y)dy \right\} \nu_t^{(i)}(d\mathbf{x}) && \text{births w/ mutations, w/o migrations} \\
&+ \iint_{\mathcal{X}} \left\{ \frac{1}{K}(\phi(\nu_t^{(i)} - \delta_{\mathbf{x}})) - \phi(\nu_t^{(i)}))\nu_t^{(i)}(dy)\, \nu_t^{(i)}(dx) \right\} && \text{deaths} \\
&+ \sum_{j \neq i} \frac{a_{i,j}}{d_j} \int_{\mathcal{X}} \mu m \left\{ \int_{\mathcal{X}} b^{(j)}(y)(\phi(\nu^{(j)} + \delta_{\mathbf{x}}) - \phi(\nu^{(j)}))\mathcal{M}(\mathbf{x}, y)dy \right\} \nu_t^{(j)}(d\mathbf{x}) && \text{migrations w/ mutations} \\
&+ \sum_{j \neq i} \frac{a_{i,j}}{d_j} \int_{\mathcal{X}} \left\{ b^{(j)}(\mathbf{x})(1-\mu)m(\phi(\nu^{(j)} + \delta_{\mathbf{x}}) - \phi(\nu^{(j)})) \right\} \nu_t^{(j)}(d\mathbf{x}). && \text{migrations w/o mutations}
\end{aligned}
\tag{S2}
$$

Taking expectations in Eq. (S2), one can obtain an equation for the mean trajectory of the quantity of interest, $\mathbb{E}\left[\phi(\nu_t^{(i)})\right]$. Nonetheless, Eq. (S2) involves an integral with respect to $\nu_t^{(i)}(dx)\nu_t^{(i)}(dy)$, making it impossible to obtain an explicit solution. It is therefore unclear whether one can gain insight into the stochastic dynamics from Eq. (S2) without simplifying assumptions. We refer to [3] for a detailed discussion on the topic.

A.2 Deterministic approximation

One strategy to overcome the difficulties encountered above is to assimilate the process to its mean trajectory, assuming that $\mathbb{E}\left[\nu_t^{(i)}\right] \approx \nu_t^{(i)}$ and further approximating $\nu_t^{(i)}$ with a continuous deterministic function $n_t^{(i)}$. Such strategy inherently neglects the stochasticity of the process, which is reasonable provided that a force dampens the stochastic fluctuations of the quantity of interest.

A.2.1 Setting with no selection

Consider a setting with no selection and recall that in this setting where $x \equiv u \in \mathcal{X} = \mathcal{U}$ we define

$$b^{(i)}(x) \equiv b \tag{S3}$$

By applying the strategy mentioned above and choosing $\phi(n_t^{(i)}) = \int_{\mathcal{X}} n_t^{(i)}(x)dx$, Eq. (S2) transforms into the deterministic approximation of the population size dynamics given in the main-text by

$$\partial_t N_t^{(i)} = N_t^{(i)} \left[b(1-m) - \frac{N_t^{(i)}}{K} \right] + mb \sum_{j \neq i} \frac{a_{i,j}}{d_j} N_t^{(j)}. \tag{S4}$$

Competition stabilises the population size dynamics, which behaves deterministically. This is supported by Supplementary Fig. 10a, which shows how Eq. (S4) accurately describes the population size for varying migration regimes. Nonetheless, stochastic fluctuations drive the dynamics of the neutral trait distribution. Attempting to characterise the neutral trait distribution with the same strategy, this

time setting $\phi(n_t^{(i)}) = n_t^{(i)}(u)$, yields

$$\begin{aligned}\partial_t n_t^{(i)}(u) = n_t^{(i)}(u) &\left[b(1-m)(1-\mu) - \frac{1}{K}\int_{\mathcal{U}} n_t^{(i)}(\mathbf{u})\,d\mathbf{u})\right] \\ &+ (1-m)\mu b \int_{\mathcal{U}} n_t^{(i)}(\mathbf{u})\mathcal{M}(u,\mathbf{u})\,d\mathbf{u} \\ &+ m\mu b \sum_{j\neq i} \frac{a_{i,j}}{d_j} \int_{\mathcal{U}} n_t^{(j)}(u)\mathcal{M}(u,\mathbf{u})d\mathbf{u} \\ &+ m(1-\mu)b \sum_{j\neq i} \frac{a_{i,j}}{d_j} b\, n_t^{(j)}(u).\end{aligned} \tag{S5}$$

Solving for Eq. (S5), one can show that the variance of $n_t^{(i)}$ continuously grows in time (see Supplementary Fig. 10) and tends to infinity as time goes to infinity, which is an unrealistic behaviour considering finite populations. Intuitively, this reflects the fact that no stabilising force acts on the neutral trait distribution, such that random fluctuations play a major role in driving the dynamics of the stochastic process. Supplementary Fig. 10 shows how IBM trajectories significantly differ from Eq. (S5), and Supplementary Fig. 11 illustrates how diversity metrics obtained from Eq. (S5) do not match those obtained from simulations of the IBM.

A.2.2 Setting with heterogeneous selection

In contrast to the neutral trait dynamics, the adaptive distribution can successfully be approximated by a deterministic description because selection pressure acts as a stabilising force and stabilises the populations' adaptive trait, dampening the stochastic fluctuations. Consider the setting with heterogeneous selection and recall that in this setting where $x \equiv (s,u) \in \mathcal{X} = \mathcal{S} \times \mathcal{U}$ we define

$$b^{(i)}(x) \equiv b(1 - p(s-\theta_i)^2). \tag{S6}$$

By applying the same strategy as above to characterise the adaptive trait distribution $n_t^{(i)}(s)$ by choosing $\phi(n_t^{(i)}) = n_t^{(i)}(s) \equiv \int_{\mathcal{U}} n_t^{(i)}(u,s)du$, Eq. (S2) transforms into

$$\begin{aligned}\partial_t n_t^{(i)}(s) = n_t^{(i)}(s) &\left[b^{(i)}(s)(1-m)(1-\mu) - \frac{1}{K}\int_{\mathcal{S}} n_t^{(i)}(\mathbf{s})\,d\mathbf{s})\right] \\ &+ (1-m)\mu \int_{\mathcal{S}} b^{(i)}(\mathbf{s})\, n_t^{(i)}(\mathbf{s})\mathcal{M}(\mathbf{s},s)\,d\mathbf{s} \\ &+ m\mu \sum_{j\neq i} \frac{a_{i,j}}{d_j} \int_{\mathbb{R}} b^{(j)}(\mathbf{s})\, n_t^{(j)}(s)\mathcal{M}(\mathbf{s},s)d\mathbf{s} \\ &+ m(1-\mu) \sum_{j\neq i} \frac{a_{i,j}}{d_j} b^{(j)}(s)\, n_t^{(j)}(s).\end{aligned} \tag{S7}$$

Assuming that the variance of the mutation kernel is small, one can use a diffusion approximation for the mutation term [4, 5, 6]

$$\int_{\mathcal{S}} b^{(i)}(\mathbf{s})\, n_t^{(i)}(\mathbf{s})\mathcal{M}(\mathbf{s},s)\,d\mathbf{s} = b^{(i)}(s,t)\, n_t^{(i)}(s) + \tfrac{1}{2}\sigma_\mu^2 \Delta_s (b^{(i)} n_t^{(i)})(s). \tag{S8}$$

Neglecting the terms in $m\mu$, we obtain

$$\begin{aligned}\partial_t n_t^{(i)}(s) = n_t^{(i)}(s)\left[b^{(i)}(s,t)(1-m-\mu) - \frac{1}{K}\int_{\mathcal{S}} n_t^{(i)}(\mathbf{s})\,d\mathbf{s}\right] \\ + \mu\left[b^{(i)}(s,t)\,n_t^{(i)}(s) + \tfrac{1}{2}\sigma_\mu^2\Delta_s(b^{(i)}n_t^{(i)})(s)\right] \\ + m\sum_{j\neq i} b^{(j)}(s,t)n_t^{(j)}(s)a_{i,j}\end{aligned} \tag{S9}$$

which, after rearranging terms, yields the elegant deterministic approximation of the adaptive trait dynamics

$$\partial_t n_t^{(i)}(s) = n_t^{(i)}(s)\left[b^{(i)}(s)(1-m) - \frac{1}{K}\int_{\mathcal{S}} n_t^{(i)}(\mathbf{s})d\mathbf{s}\right] + m\sum_{j\neq i} b^{(j)}(s)\frac{a_{i,j}}{d_j}n_t^{(j)}(s) + \tfrac{1}{2}\mu\sigma_\mu^2\Delta_s\left[b^{(i)}(s)n_t^{(i)}(s)\right]. \tag{S10}$$

Setting $m = 0$ [6] shows that Eq. (S10) admits a stationary solution that is Gaussian, with variance $\sqrt{\mu}\sigma_\mu^2/\sqrt{p}$. Therefore, the variance of the adaptive trait distribution stabilises to a finite value. Intuitively, this reflects the fact that the random fluctuations of the adaptive trait distribution are dampened by the stabilising force of selection. Provided that the selection strength p is large enough, Eq. (S10) is a good approximation of the adaptive trait distribution obtained from the stochastic process. Supplementary Fig. 3 shows how IBM trajectories are similar to the ones obtained from Eq. (S5), and Supplementary Fig. 4 illustrates how diversity metrics obtained from Eq. (S5) match those obtained from simulations of the IBM.

A.3 Trait-dependent competition

To test whether the effects of the metrics hold under more complex ecological processes, we designed an extra experiment considering heterogeneous selection and adaptive trait-dependent competition, where the death rate of individuals on v_i with traits $x_k^{(i)} = (u_k^{(i)}, s_k^{(i)}) \in \mathcal{U}\times\mathcal{S}$ is given by

$$d(x_k^{(i)}, \nu^{(i)}) = \frac{1}{K}\int_{\mathcal{S}} \exp\Big(-\frac{(s_k^{(i)} - \mathbf{s})^2}{2\sigma_\alpha^2}\Big)\nu^{(i)}(\mathbf{s}) \tag{S11}$$

where σ_α is the competition bandwidth. This competition kernel tends to increase the population size, as it decreases the overall competition. The adaptive dynamics theory predicts that when $m = 0$, competition promotes two distinct types of individuals at either side of the adaptive trait optimum for a competition bandwidth $\sigma_\alpha < 1/\sqrt{2p}$, while a single type is observed when $\sigma_\alpha > 1/\sqrt{2p}$ [7]. We performed simulations in both cases for graphs with $M = 7$ vertices and show results of the multivariate regression analyses in Supplementary Table 5. The analyses demonstrate that the trends reported in the main manuscript remain unchanged in both cases.

A.4 Derivation of the habitat assortativity metric r_Θ in binary environments

We demonstrate here how the habitat assortativity r_Θ relates to the conditional probability of habitats being connected, and we show how r_Θ simplifies under mean field assumption.

Following the original definition of [8], habitat assortativity r_Θ is defined as the Pearson correlation of environmental conditions θ at either ends of the vertices V of graph G, that is

$$r_\Theta = \frac{\mathrm{Cov}(\Theta_\times, \Theta_\wedge)}{\sqrt{\mathrm{Var}(\Theta_\times)\mathrm{Var}(\Theta_\wedge)}} = \frac{\langle\Theta_\times\Theta_\wedge\rangle - \langle\Theta_\times\rangle\langle\Theta_\wedge\rangle}{\sqrt{(\langle\Theta_\times^2\rangle - \langle\Theta_\times\rangle^2)(\langle\Theta_\wedge^2\rangle - \langle\Theta_\wedge\rangle^2}} \tag{S12}$$

where $\Theta_\times$ and $\Theta_\wedge$ denote the sets of environmental conditions found at the toe and tip of each directed vertex of graph V, and $\langle\Theta_\times\rangle$ and $\langle\Theta_\wedge\rangle$ denote their respective mean values.

Let $P(\mathbf{II},\mathbf{I})$ be the proportion of edges that connect a vertex of habitat type $\mathbf{I}$ to a vertex of habitat type $\mathbf{II}$. One can also view $P(\mathbf{II},\mathbf{I})$ as the conditional probability that a vertex of type $\mathbf{I}$ is connected to a vertex of type $\mathbf{II}$. Let $P(\mathbf{II})$ denote the proportion of vertices that are of type $\mathbf{II}$. First observe that for undirected graphs, one has $\langle\Theta_\times\rangle = \langle\Theta_\wedge\rangle$ and $\langle\Theta_\times^2\rangle = \langle\Theta_\wedge^2\rangle$. Assuming that habitats are symmetric and binary, it follows that $\theta_{\mathbf{II}} = -\theta_{\mathbf{I}}$. Then

$$\begin{aligned}\langle\Theta_\times\Theta_\wedge\rangle &= P(\mathbf{I},\mathbf{I})\theta_{\mathbf{I}}^2 + P(\mathbf{II},\mathbf{II})\theta_{\mathbf{II}}^2 + [P(\mathbf{II},\mathbf{I}) + P(\mathbf{I},\mathbf{II})]\theta_{\mathbf{I}}\theta_{\mathbf{II}} \\ &= \theta_{\mathbf{II}}^2\left(P(\mathbf{I},\mathbf{I}) + P(\mathbf{II},\mathbf{II}) - [P(\mathbf{II},\mathbf{I}) + P(\mathbf{I},\mathbf{II})]\right),\end{aligned} \tag{S13}$$

$$\begin{aligned}\langle\Theta_\times\rangle &= P(\mathbf{I})\theta_{\mathbf{I}} + P(\mathbf{II})\theta_{\mathbf{II}} \\ &= \theta_{\mathbf{II}}\left[P(\mathbf{II}) - P(\mathbf{I})\right],\end{aligned} \tag{S14}$$

$$\begin{aligned}\langle\Theta_\times^2\rangle &= P(\mathbf{I})\theta_{\mathbf{I}}^2 + P(\mathbf{II})\theta_{\mathbf{II}}^2 \\ &= \theta_{\mathbf{II}}^2\left[P(\mathbf{I}) + P(\mathbf{II})\right] \\ &= \theta_{\mathbf{II}}^2.\end{aligned} \tag{S15}$$

Combining Eq. (S13), Eq. (S14) and Eq. (S15) with Eq. (S12) one gets

$$\begin{aligned}r_\Theta &= \frac{\langle\Theta_\times\Theta_\wedge\rangle - \langle\Theta_\times\rangle\langle\Theta_\wedge\rangle}{\langle\Theta_\times^2\rangle - \langle\Theta_\times\rangle^2} \\ &= \frac{P(\mathbf{I},\mathbf{I}) + P(\mathbf{II},\mathbf{II}) - [P(\mathbf{II},\mathbf{I}) + P(\mathbf{I},\mathbf{II})] - (P(\mathbf{II}) - P(\mathbf{I}))^2}{P(\mathbf{I}) + P(\mathbf{II}) - (P(\mathbf{II}) - P(\mathbf{I}))^2} \\ &= \frac{P(\mathbf{I},\mathbf{I}) + P(\mathbf{II},\mathbf{II}) - [P(\mathbf{II},\mathbf{I}) + P(\mathbf{I},\mathbf{II})] - (P(\mathbf{II}) - P(\mathbf{I}))^2}{1 - (P(\mathbf{II}) - P(\mathbf{I}))^2}.\end{aligned} \tag{S16}$$

Assuming that habitats are homogeneously distributed, we have $P(\mathbf{I}) = P(\mathbf{II}) = \frac{1}{2}$ and thus we obtain

$$r_\Theta = P(\mathbf{I},\mathbf{I}) + P(\mathbf{II},\mathbf{II}) - [P(\mathbf{II},\mathbf{I}) + P(\mathbf{I},\mathbf{II})]. \tag{S17}$$

The mean field approximation involves the assumption that all vertices with similar habitats are equivalent in terms of their connections with other habitats, so that $P(\mathbf{I},\mathbf{I}) = P(\mathbf{II},\mathbf{II})$ and $P(\mathbf{II},\mathbf{I}) = P(\mathbf{I},\mathbf{II})$, which yields $r_\Theta = 2\left(P(\mathbf{I},\mathbf{I}) - P(\mathbf{I},\mathbf{II})\right)$.

Supplementary References

[1] Vincent Bansaye and Sylvie Méléard. Some stochastic models for structured populations : scaling limits and long time behavior. *Stochastic Models for Structured Populations: Scaling Limits and Long Time Behavior*, pages 1–107, jun 2015.

[2] Heiner Linke. Applications of Brownian Motion. 5114:199–213, aug 2015.

[3] Nicolas Champagnat, Régis Ferrière, and Sylvie Méléard. Unifying evolutionary dynamics: From individual stochastic processes to macroscopic models. *Theoretical Population Biology*, 69(3):297–321, may 2006.

[4] M. Kimura. A stochastic model concerning the maintenance of genetic variability in quantitative characters. *Proceedings of the National Academy of Sciences of the United States of America*, 54(3):731–736, sep 1965.

[5] F. Débarre, O. Ronce, and S. Gandon. Quantifying the effects of migration and mutation on adaptation and demography in spatially heterogeneous environments. *Journal of Evolutionary Biology*, 26(6):1185–1202, 2013.

[6] Sepideh Mirrahimi and Sylvain Gandon. Evolution of specialization in heterogeneous environments: equilibrium between selection, mutation and migration. *Genetics*, 214(2):479–491, feb 2020.

[7] Michael Doebeli. *Adaptive diversification.* Monographs in population biology. Princeton University Press, Princeton, N.J, 2011.

[8] M. E. J. Newman. Mixing patterns in networks. *Physical Review E*, 67(2):026126, feb 2003.

[9] Wen-Na Ding, Richard H. Ree, Robert A. Spicer, and Yao-Wu Xing. Ancient orogenic and monsoon-driven assembly of the world's richest temperate alpine flora. *Science*, 369(6503):578–581, jul 2020.

[10] Martin Jung, Prabhat Raj Dahal, Stuart H. M. Butchart, Paul F. Donald, Xavier De Lamo, Myroslava Lesiv, Valerie Kapos, Carlo Rondinini, and Piero Visconti. A global map of terrestrial habitat types. *Scientific Data*, 7(1):256, dec 2020.

[11] Dirk Nikolaus Karger, Olaf Conrad, Jürgen Böhner, Tobias Kawohl, Holger Kreft, Rodrigo Wilber Soria-Auza, Niklaus E. Zimmermann, H. Peter Linder, and Michael Kessler. Climatologies at high resolution for the earth's land surface areas. *Scientific Data*, 4(1):170122, dec 2017.

B Supplementary Figures

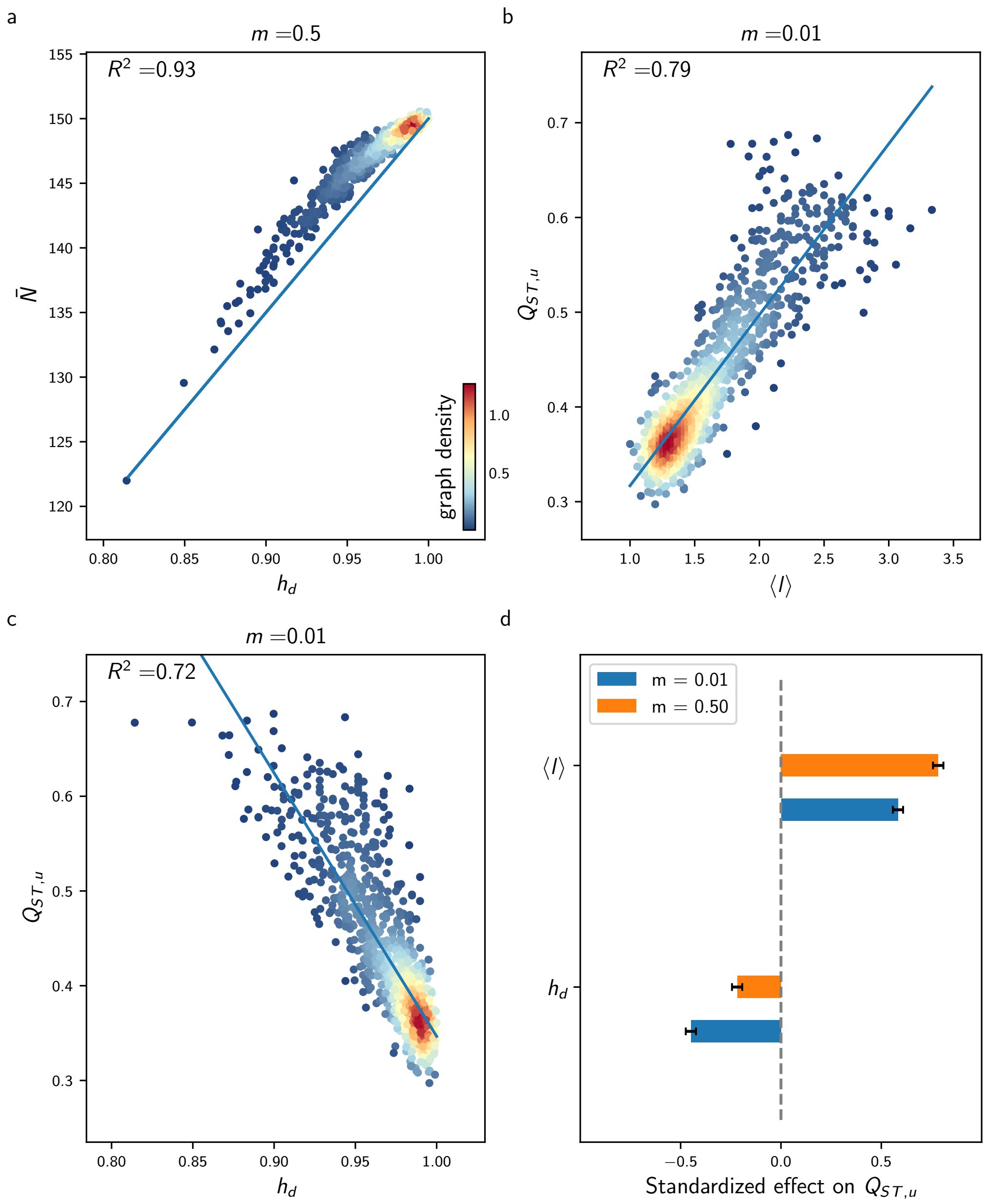

Supplementary Figure 1: Effect of $\langle l \rangle$ and h_d on average population size $\bar{N}$ and neutral differentiation $Q_{ST,u}$ under the setting with no selection, analogous to Fig. 2 but for 1126 of the 261,080 undirected connected graphs with $M = 9$ vertices.

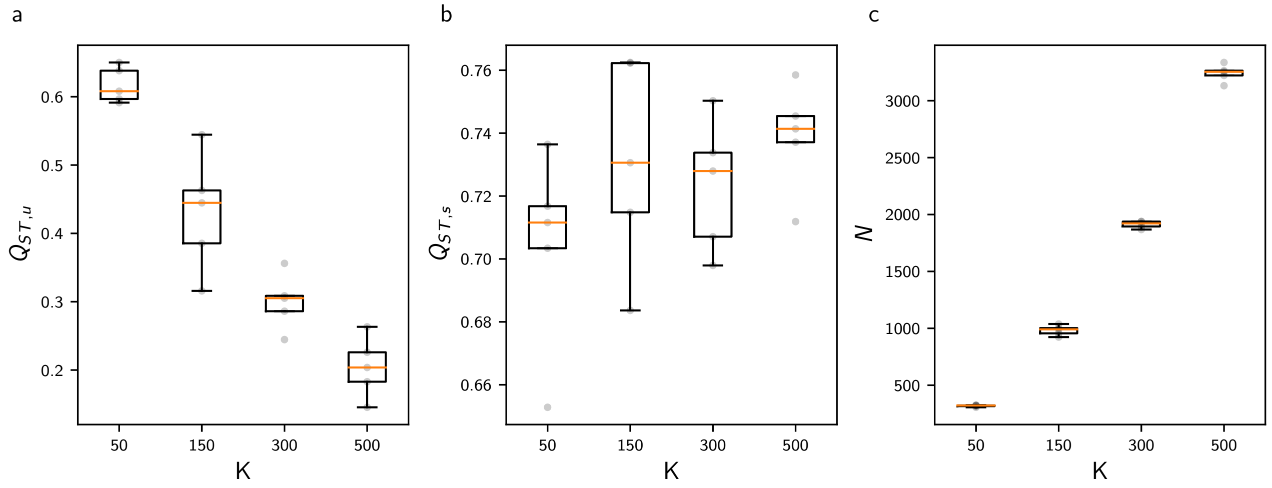

Supplementary Figure 2: Effect of the carrying capacity K on $Q_{ST,u}$, $Q_{ST,s}$ and metapopulation size N for the line graph with $M = 7$ vertices for $m = 0.1$. Decreasing K increases $Q_{ST,u}$ as it favours drift, but it does not influence $Q_{ST,s}$. Each boxplot is based on 5 replicate simulations of the IBM, and fade dots represent single values for each replicate.

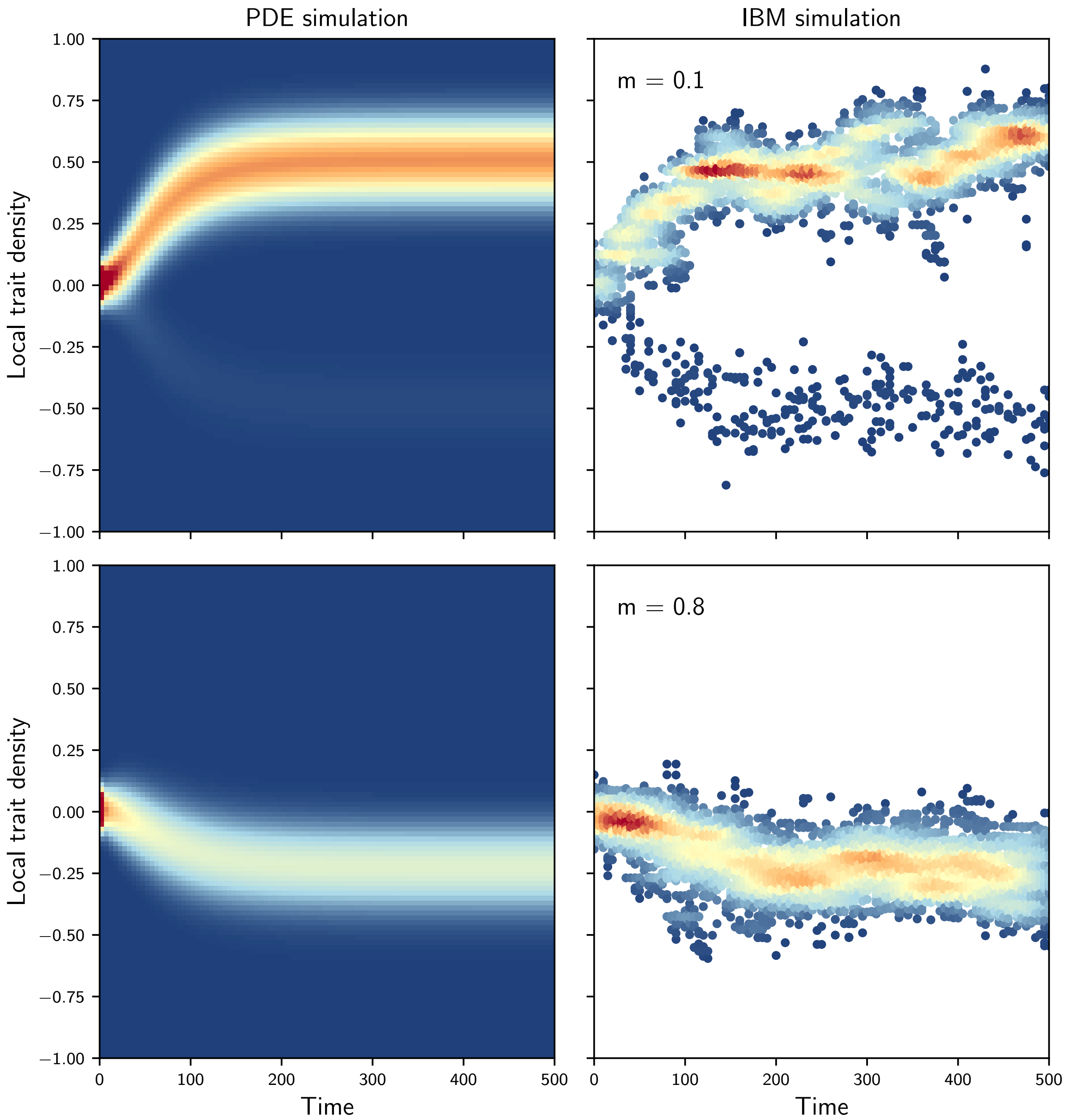

Supplementary Figure 3: Comparison of the adaptive trait density on one vertex obtained from Eq. (S10) (left) and from the IBM simulations (right) in the setting with heterogeneous selection, for the star graph with $M = 7$ vertices. The densities obtained from Eq. (S10) and from the IBM are qualitatively similar.

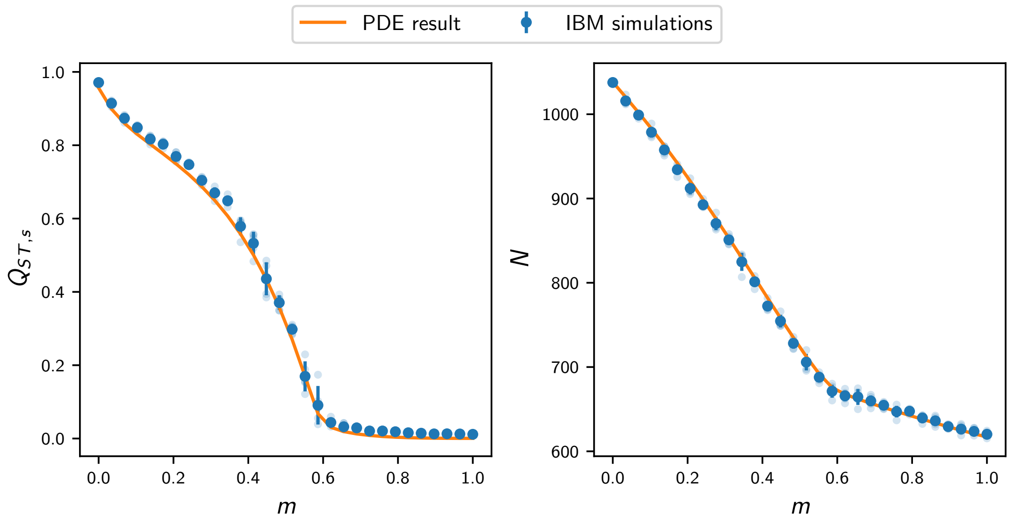

Supplementary Figure 4: Comparison of $Q_{ST,s}$ and N obtained from the deterministic approximation Eq. (S10) and from IBM simulations in the setting with heterogeneous selection, on the star graph with $M = 7$ vertices. $Q_{ST,s}$ and population size obtained from Eq. (S10) closely match the IBM simulations. Each plain dot represents average results from 5 replicate simulations of the IBM, bars represent one standard deviation, and each fade dot represents a single replicate value.

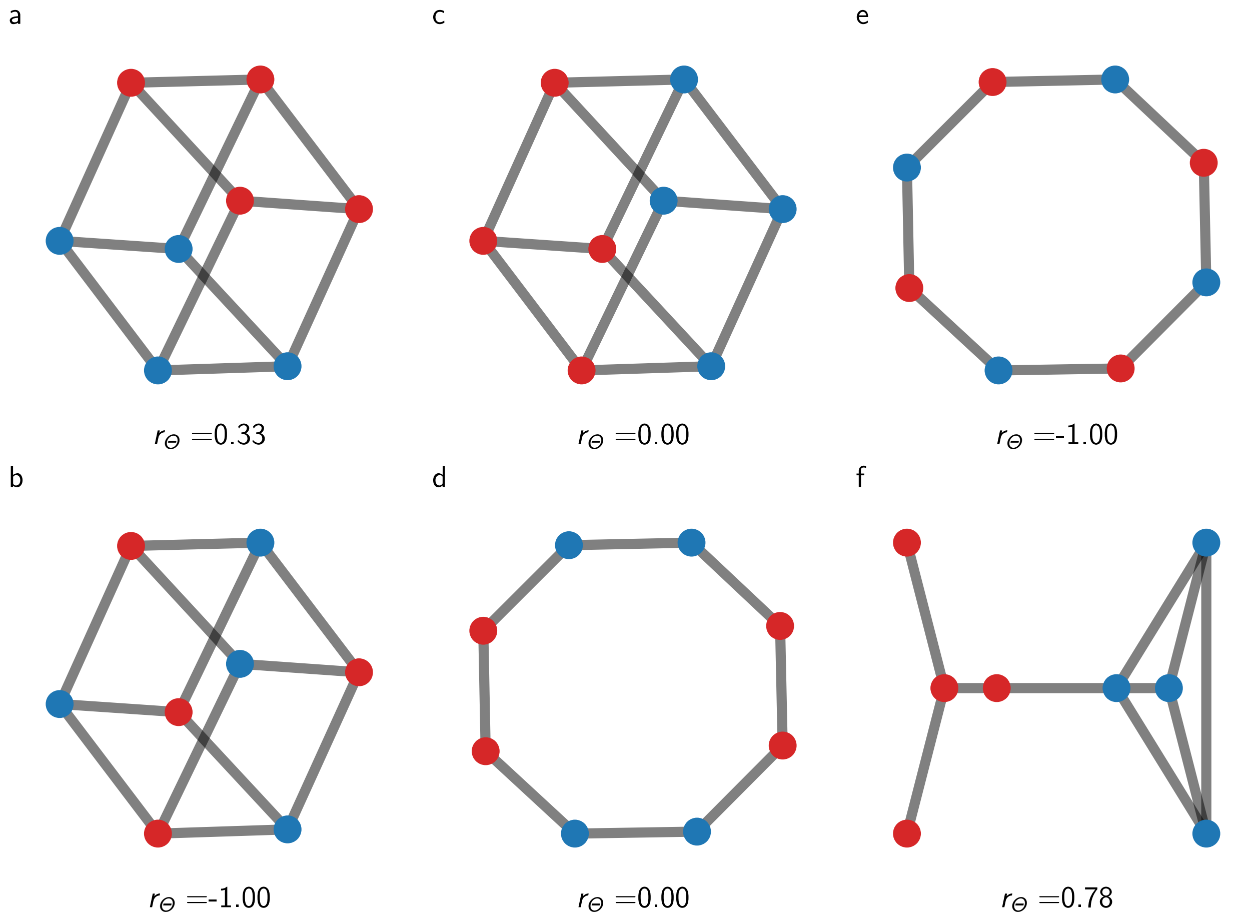

Supplementary Figure 5: Graphs with spatial distribution of habitat types corresponding to different habitat assortativity r_Θ. Graphs (a–d) can be described exactly with a mean field approach, as blue and red vertices have an equivalent position on the graph.

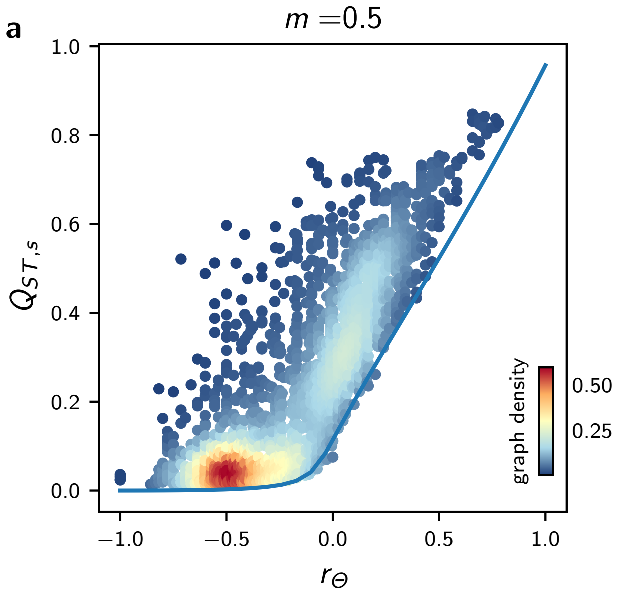

Supplementary Figure 6: Effects of habitat heterogeneity r_Θ on $Q_{ST,s}$ and average population size $\bar{N}$ for all undirected connected graphs with $M = 7$ vertices and varying r_Θ, obtained for similar simulations to those in Fig. 4 with $m = 0.5$. In (a) and (b), each dot represents average results from 5 replicate simulations of the IBM, the colour scale corresponds to the proportion of the graph with similar x and y axis values (graph density), and the blue lines correspond to results obtained from the mean field, deterministic approximation Eq. (5). Deviations from the mean field, deterministic approximation Eq. (5) can be explained by differences in $\langle l \rangle$ and h_d between the graphs.

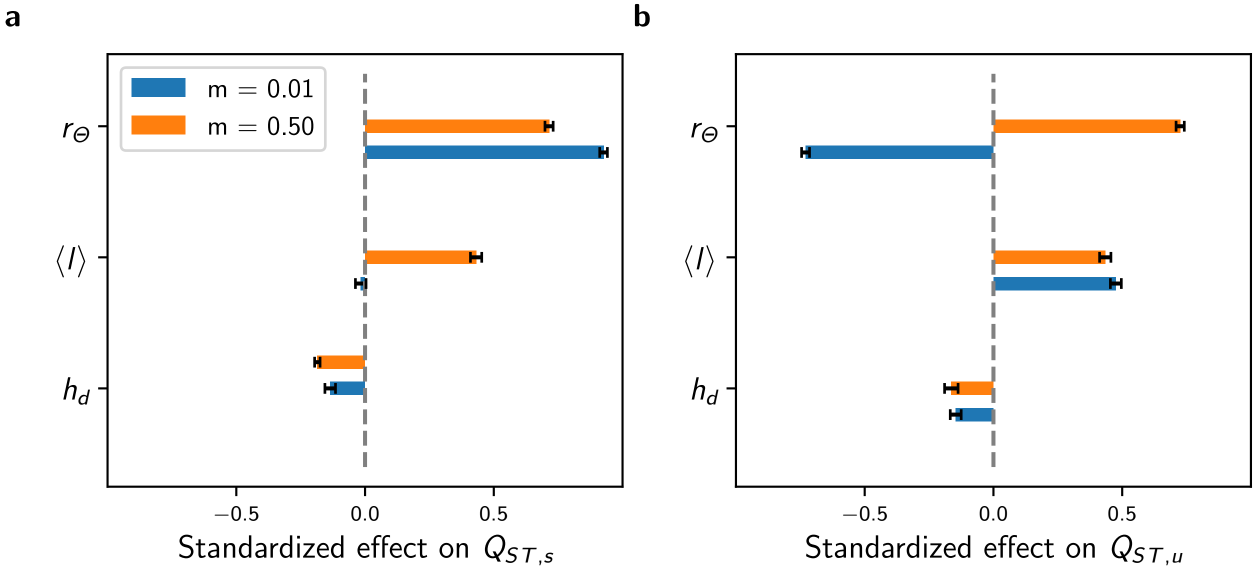

Supplementary Figure 7: Standardized effects of h_d, $\langle l \rangle$ and r_Θ on $Q_{ST,s}$ and $Q_{ST,u}$ obtained from multivariate regression models independently fitted for low and high migration regimes on average results from 5 replicate simulations of the IBM, analogous Fig. 5c–d but for 1126 of the 261,080 undirected connected graphs with $M = 9$ vertices and varying r_Θ (see Methods for details). Error bars show 95% confidence intervals.

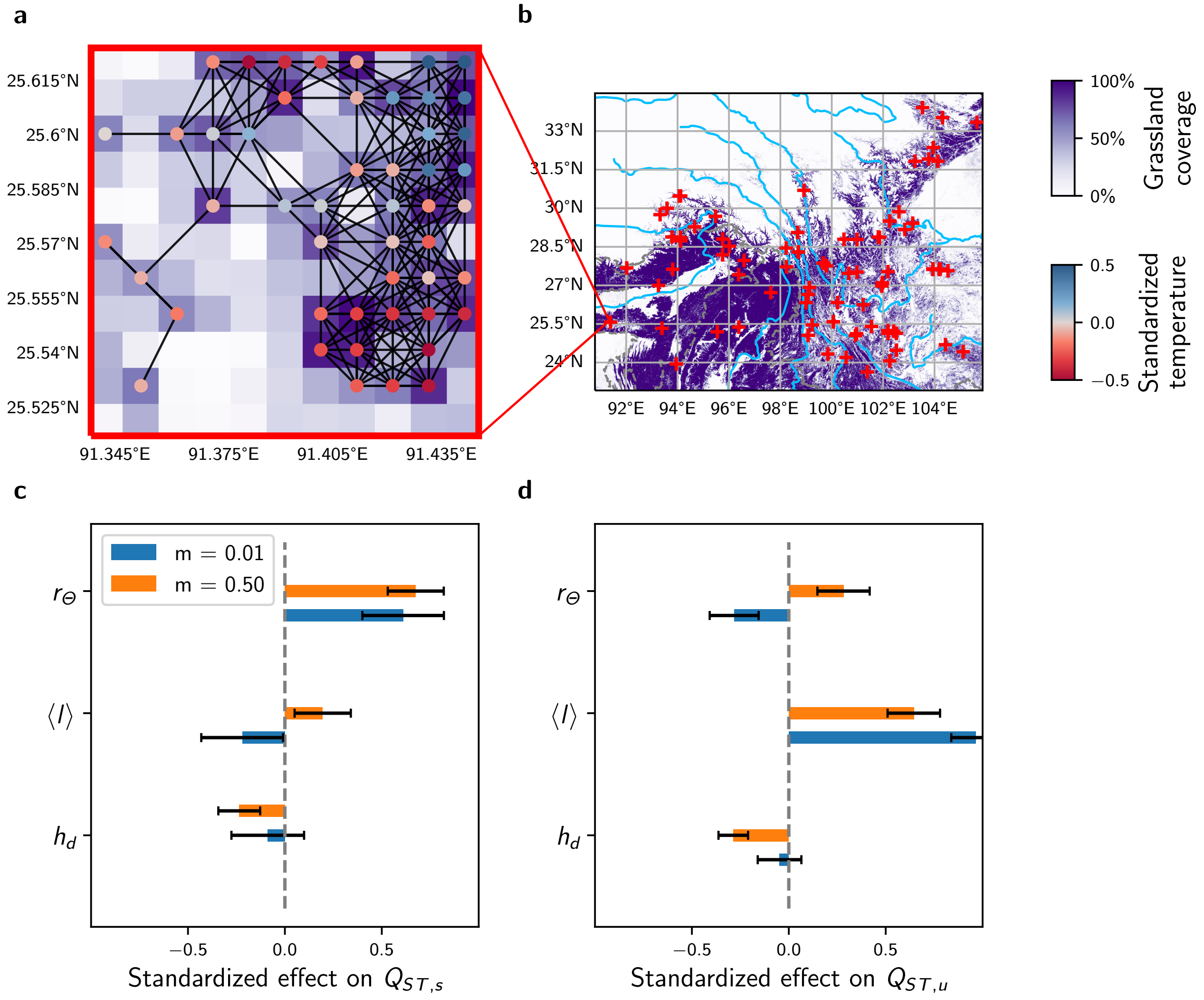

Supplementary Figure 8: Simulations on graphs with $M = 49$ vertices obtained from real spatial habitat datasets, in the setting with heterogeneous selection. The region from where graphs are obtained is centred on the Hengduan Mountains in Southwest China, one of the most species-rich temperate mountain biota globally [9]. (a) Graphical representation of a geographical area of size $0.11° \times 0.11°$. To create the graph, we considered biological populations living in grasslands, and used the dataset provided in [10] containing global grassland coverage at 0.01° resolution. We assigned a vertex to a geographical area of size $0.01° \times 0.01°$ if its grassland coverage was above a threshold arbitrarily set to 50%. We further assumed that two vertices were connected if their euclidean distance was below a certain dispersal range, which we let vary from 1 to 2.5 km. Local annual average temperature was considered as the value that captures environmental conditions at each vertex. Temperature data was obtained from the CHELSA dataset [11]. (b) Grassland coverage for the considered region. Blue lines correspond to rivers and dashed grey lines correspond to country borders. Red crosses indicate the locations of the 83 graphs sampled for the simulations used in (c–d). (c–d) Standardized effects of h_d, $\langle l \rangle$ and r_Θ on $Q_{ST,s}$ and $Q_{ST,u}$ obtained from multivariate regression models independently fitted for low and high migration regimes to average results from 5 replicate simulations of the IBM on the 83 graphs which location is illustrated in (c) (see Supplementary Table 4 for simulation details). Error bars show 95% confidence intervals.

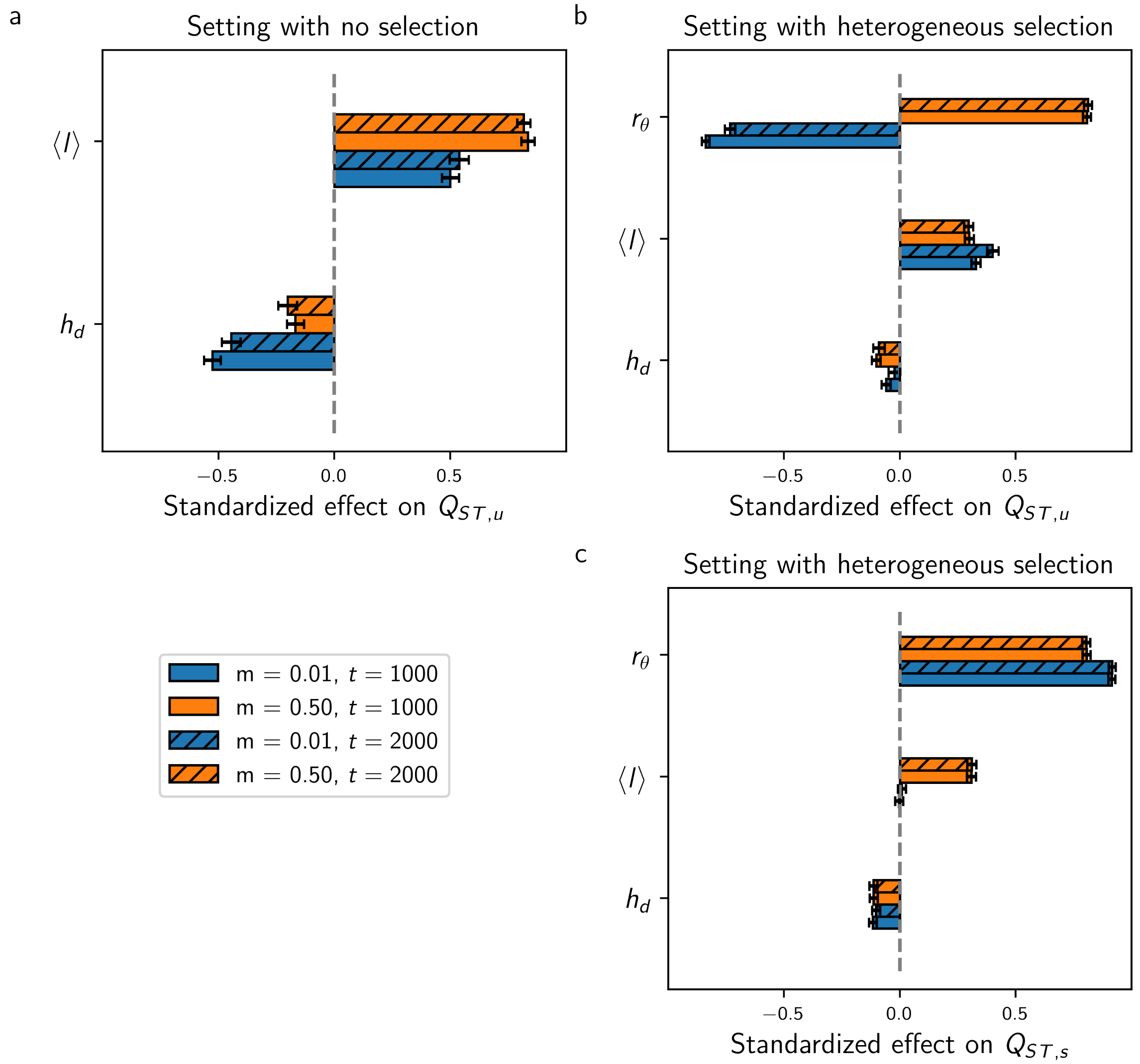

Supplementary Figure 9: Standardized effects of h_d, $\langle l \rangle$ and r_Θ on $Q_{ST,u}$ in the setting with no selection and in the setting with heterogeneous selection for the time horizons $t = 1000$ and $t = 2000$, obtained from multivariate regression models independently fitted for low and high migration regimes to average results from 5 replicate simulations of the IBM on all undirected connected graphs with $M = 7$ vertices and varying r_Θ (see Methods for details). (a–c) illustrate that the effects of the topology metrics on $Q_{ST,u}$ and $Q_{ST,s}$ remain constant for $t > 1000$ in both the settings without selection and with heterogeneous selection. Error bars show 95% confidence intervals.

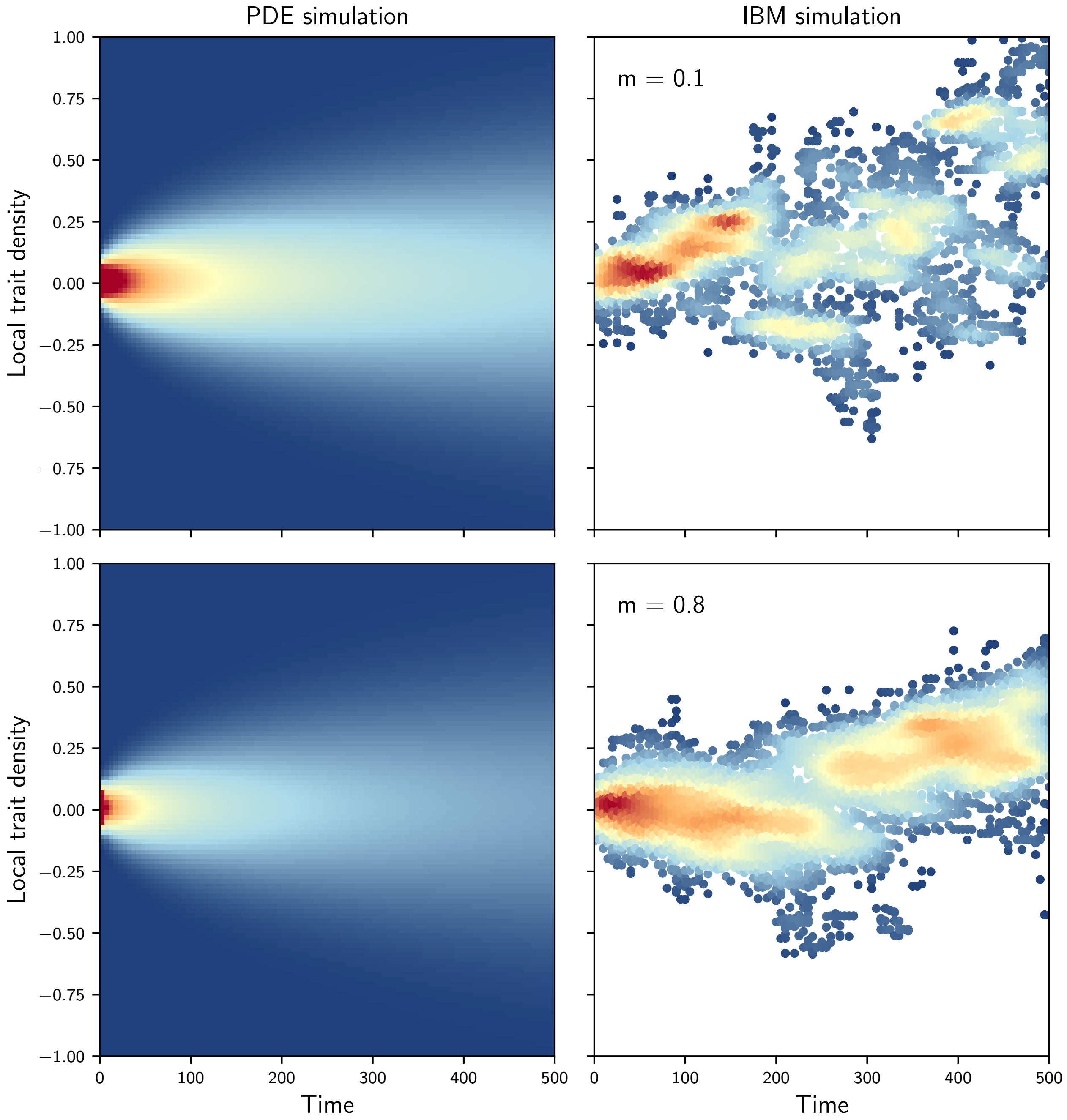

Supplementary Figure 10: Comparison of the neutral trait density on one vertex obtained from Eq. (S5) (left) and from the IBM simulations (right) in the setting with no selection, for the chain graph. The densities obtained from Eq. (S5) and from the IBM are dissimilar.

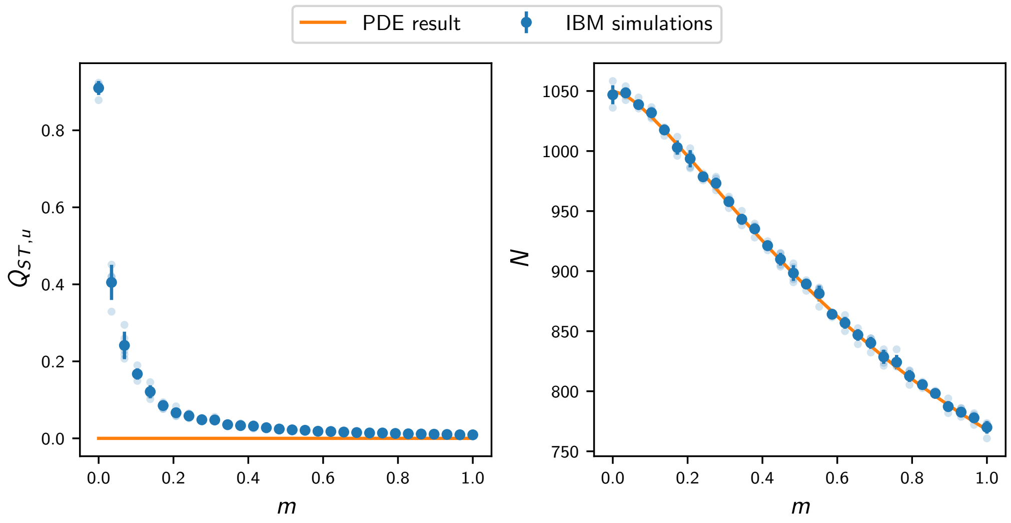

Supplementary Figure 11: Comparison of results obtained from the deterministic approximations Eqs. (S4) and (S5) and from IBM simulations in the setting with no selection, on the star graph with $M = 7$ vertices. While Eq. (S4) can capture population size, Eq. (S5) is not able to capture $Q_{ST,u}$. Each plain dot represents average results from 5 replicate simulations, bars represent one standard deviation, and each fade dot represents a single replicate value.

C Supplementary Tables

Supplementary Table 1: Linear regression model coefficients for the effect of topology metrics on $Q_{ST,u}$ in the setting with no selection, based on all graphs with $M = 7$ vertices. *** $P < 0.001$

	$Q_{ST,u}$				$Q_{ST,u} - bN$	
m	0.01	0.50	0.01	0.50	0.01	0.50
(Intercept)	0.000	-0.000	-0.000	-0.000	-0.000	-0.000
	(0.023)	(0.017)	(0.023)	(0.025)	(0.023)	(0.028)
$\langle l \rangle$	0.739***	0.872***				
	(0.023)	(0.017)				
h_d			-0.753***	-0.674***	-0.753***	-0.143***
			(0.023)	(0.025)	(0.023)	(0.028)
Number of sim.	853	853	853	853	853	853
R^2	0.546	0.760	0.567	0.454	0.567	0.030

Supplementary Table 2: Multivariate linear regression model coefficients for the effect of topology metrics on $Q_{ST,u}$ in the setting with no selection. *** $P < 0.001$

	$M = 7$		$M = 9$	
	$Q_{ST,u}$			
m	0.01	0.50	0.01	0.50
(Intercept)	-0.000	-0.000	0.000	-0.000
	(0.017)	(0.013)	(0.009)	(0.010)
h_d	-0.527***	-0.352***	-0.449***	-0.218***
	(0.019)	(0.014)	(0.013)	(0.013)
$\langle l \rangle$	0.500***	0.712***	0.583***	0.784***
	(0.019)	(0.014)	(0.013)	(0.013)
Number of sim.	853	853	1,126	1,126
R^2	0.766	0.858	0.899	0.896

Supplementary Table 3: Multivariate linear regression model coefficients for the effect of the topology metrics on $Q_{ST,u}$ and $Q_{ST,s}$ in the setting with heterogeneous selection. *** $P < 0.001$

	$M = 7$				$M = 9$			
	$Q_{ST,s}$		$Q_{ST,u}$		$Q_{ST,s}$		$Q_{ST,u}$	
m	0.01	0.50	0.01	0.50	0.01	0.50	0.01	0.50
(Intercept)	-0.000	-0.000	-0.000	-0.000	0.000	0.000	0.000	0.000
	(0.008)	(0.009)	(0.009)	(0.009)	(0.008)	(0.008)	(0.008)	(0.008)
h_d	-0.117***	-0.114***	-0.060***	-0.102***	-0.135***	-0.185***	-0.146***	-0.164***
	(0.009)	(0.010)	(0.010)	(0.010)	(0.010)	(0.011)	(0.011)	(0.011)
$\langle l \rangle$	-0.004	0.308***	0.328***	0.300***	-0.017	0.431***	0.475***	0.434***
	(0.009)	(0.010)	(0.010)	(0.010)	(0.010)	(0.011)	(0.011)	(0.011)
r_Θ	0.914***	0.805***	-0.838***	0.807***	0.926***	0.715***	-0.730***	0.725***
	(0.008)	(0.009)	(0.009)	(0.009)	(0.008)	(0.008)	(0.008)	(0.008)
Number of sim.	2,548	2,548	2,548	2,548	2,250	2,250	2,250	2,250
R^2	0.845	0.808	0.808	0.799	0.870	0.853	0.862	0.851

Supplementary Table 4: Multivariate linear regression model coefficients for the effect of topology metrics on $Q_{ST,u}$ and $Q_{ST,s}$ on real graphs with $M = 49$ vertices in the setting with heterogeneous selection. * $P < 0.05$, ** $P < 0.01$, *** $P < 0.001$

	$Q_{ST,s}$		$Q_{ST,u}$	
m	0.1	0.50	0.1	0.50
(Intercept)	-0.000	-0.000	0.000	-0.000
	(0.093)	(0.064)	(0.056)	(0.059)
h_d	-0.088	-0.235***	-0.048	-0.286***
	(0.094)	(0.065)	(0.057)	(0.060)
$\langle l \rangle$	-0.220*	0.195**	0.965***	0.645***
	(0.106)	(0.073)	(0.064)	(0.068)
r_Θ	0.610***	0.675***	-0.282***	0.282***
	(0.106)	(0.073)	(0.063)	(0.068)
Number of sim.	83	83	83	83
R^2	0.313	0.675	0.752	0.717

Supplementary Table 5: Multivariate linear regression model coefficients for the effect of topology metrics on $Q_{ST,u}$ and $Q_{ST,s}$ in the setting of trait-dependent competition and heterogeneous selection (Section A.3), based on all graphs with $M = 7$ vertices. *** $P < 0.001$

	$\sigma_a = 0.5 < 1/\sqrt{2p}$				$\sigma_a = 1 > 1/\sqrt{2p}$			
	$Q_{ST,s}$		$Q_{ST,u}$		$Q_{ST,s}$		$Q_{ST,u}$	
m	0.05	0.50	0.05	0.50	0.05	0.50	0.05	0.50
(Intercept)	0.000	-0.000	-0.000	-0.000	0.000	-0.000	0.000	-0.000
	(0.005)	(0.010)	(0.011)	(0.010)	(0.004)	(0.008)	(0.012)	(0.007)
h_d	-0.228***	-0.118***	-0.171***	-0.169***	-0.166***	-0.128***	-0.178***	-0.139***
	(0.006)	(0.011)	(0.012)	(0.012)	(0.004)	(0.009)	(0.013)	(0.008)
$\langle l \rangle$	0.084***	0.373***	0.461***	0.573***	0.002	0.296***	0.483***	0.286***
	(0.006)	(0.011)	(0.012)	(0.012)	(0.004)	(0.009)	(0.013)	(0.008)
r_Θ	0.922***	0.741***	-0.657***	0.508***	0.967***	0.816***	-0.585***	0.837***
	(0.005)	(0.010)	(0.011)	(0.010)	(0.004)	(0.008)	(0.012)	(0.007)
Number of sim.	2,548	2,548	2,548	2,548	2,548	2,548	2,548	2,548
R^2	0.934	0.768	0.716	0.732	0.962	0.828	0.659	0.861
