# Supplementary material for: Eco-evolutionary model on spatial graphs reveals how habitat structure affects phenotypic differentiation
Source: Commun Biol. 2022 Jul 6;5:668. doi: 10.1038/s42003-022-03595-3 (PMC9259634; doi:10.1038/s42003-022-03595-3)
Supplement: Supplementary file 2 — Reporting Summary [file 42003_2022_3595_MOESM2_ESM.pdf]

nature portfolio

Corresponding author(s): Victor Boussange & Loïc Pellissier

Last updated by author(s): Jun 11, 2022

Reporting Summary

Nature Portfolio wishes to improve the reproducibility of the work that we publish. This form provides structure for consistency and transparency in reporting. For further information on Nature Portfolio policies, see our Editorial Policies and the Editorial Policy Checklist.

Statistics

For all statistical analyses, confirm that the following items are present in the figure legend, table legend, main text, or Methods section.

n/a	Confirmed	
☐	☒	The exact sample size (n) for each experimental group/condition, given as a discrete number and unit of measurement
☐	☒	A statement on whether measurements were taken from distinct samples or whether the same sample was measured repeatedly
☐	☒	The statistical test(s) used AND whether they are one- or two-sided<br>*Only common tests should be described solely by name; describe more complex techniques in the Methods section.*
☐	☒	A description of all covariates tested
☐	☒	A description of any assumptions or corrections, such as tests of normality and adjustment for multiple comparisons
☐	☒	A full description of the statistical parameters including central tendency (e.g. means) or other basic estimates (e.g. regression coefficient) AND variation (e.g. standard deviation) or associated estimates of uncertainty (e.g. confidence intervals)
☐	☒	For null hypothesis testing, the test statistic (e.g. F, t, r) with confidence intervals, effect sizes, degrees of freedom and P value noted<br>*Give P values as exact values whenever suitable.*
☐	☒	For Bayesian analysis, information on the choice of priors and Markov chain Monte Carlo settings
☐	☒	For hierarchical and complex designs, identification of the appropriate level for tests and full reporting of outcomes
☐	☒	Estimates of effect sizes (e.g. Cohen's d, Pearson's r), indicating how they were calculated

Our web collection on statistics for biologists contains articles on many of the points above.

Software and code

Policy information about availability of computer code

Data collection	The simulation code is available at https://github.com/vboussange/differentiation-in-spatial-graphs.
Data analysis	Statistical anyalses were conducted in Julia using StatsKit.jl.

For manuscripts utilizing custom algorithms or software that are central to the research but not yet described in published literature, software must be made available to editors and reviewers. We strongly encourage code deposition in a community repository (e.g. GitHub). See the Nature Portfolio guidelines for submitting code & software for further information.

Data

Policy information about availability of data

All manuscripts must include a data availability statement. This statement should provide the following information, where applicable:

- Accession codes, unique identifiers, or web links for publicly available datasets
- A description of any restrictions on data availability
- For clinical datasets or third party data, please ensure that the statement adheres to our policy

The data underlying our figures is available at https://github.com/vboussange/differentiation-in-spatial-graphs.

Field-specific reporting

Please select the one below that is the best fit for your research. If you are not sure, read the appropriate sections before making your selection.

☐ Life sciences ☐ Behavioural & social sciences ☒ Ecological, evolutionary & environmental sciences

For a reference copy of the document with all sections, see nature.com/documents/nr-reporting-summary-flat.pdf

Ecological, evolutionary & environmental sciences study design

All studies must disclose on these points even when the disclosure is negative.

Study description	Simulation study of an individual based model, complemented by analytical insights into the dynamics with a deterministic approximation.
Research sample	Simulation outputs from the individual based model, run on graphs with different topologies.
Sampling strategy	We ran simulations on all of the 853 undirected connected graphs with M=7 vertices and on 1126 of the 261,080 undirected connected graphs with M=9 vertices, listed at http://oeis.org/A001349. Graphs with M = 9 vertices were selected with a stratified sampling method: we randomly sampled without replacement a maximum of 50 graphs for each class of graphs with an equal number of vertices. For the setting with heterogeneous selection, we generated the labeled graphs by randomly generating \Theta-spatial distributions, and by using a stratified sampling strategy to select without replacement at most 3 and 2 \Theta-spatial distributions corresponding to the quartiles of the r_\theta values obtained, respectively for graphs with M=7 and M=9 vertices. This sampling strategy allowed to obtain a uniform distribution of the topology metrics investigated in the study, and therefore permitted to correctly represent the population of graphs to investigate their effect on differentiation.
Data collection	N/A
Timing and spatial scale	N/A
Data exclusions	N/A
Reproducibility	All simulations can be exactly reproduced from the code available at https://github.com/vboussange/differentiation-in-spatial-graphs.
Randomization	N/A
Blinding	N/A

Did the study involve field work? ☐ Yes ☒ No

Reporting for specific materials, systems and methods

We require information from authors about some types of materials, experimental systems and methods used in many studies. Here, indicate whether each material, system or method listed is relevant to your study. If you are not sure if a list item applies to your research, read the appropriate section before selecting a response.

Materials & experimental systems

n/a	Involved in the study	
☒	☐	Antibodies
☒	☐	Eukaryotic cell lines
☒	☐	Palaeontology and archaeology
☒	☐	Animals and other organisms
☒	☐	Human research participants
☒	☐	Clinical data
☒	☐	Dual use research of concern

Methods

n/a	Involved in the study	
☒	☐	ChIP-seq
☒	☐	Flow cytometry
☒	☐	MRI-based neuroimaging
